# Physical, Chemical, and Electrochemical Properties of Redox-Responsive Polybenzopyrrole as Electrode Material for Faradaic Energy Storage

**DOI:** 10.3390/polym13172883

**Published:** 2021-08-27

**Authors:** Bushra Begum, Salma Bilal, Anwar ul Haq Ali Shah, Philipp Röse

**Affiliations:** 1 National Centre of Excellence in Physical Chemistry, University of Peshawar, Peshawar 25120, Pakistan; bushrachemist248@gmail.com; 2 Karlsruhe Institute of Technology (KIT), Institute for Applied Materials—Electrochemical Technologies (IAM-ET), 76131 Karlsruhe, Germany; 3 Institute of Chemical Science, University of Peshawar, Peshawar 25120, Pakistan; anwarulhaqalishah@uop.edu.pk

**Keywords:** polybenzopyrrole, conductive polymers, energy storage, thermal stability, polymer electrode material

## Abstract

Polybenzopyrrole (Pbp) is an emerging candidate for electrochemical energy conversion and storage. There is a need to develop synthesis strategies for this class of polymers that can help improve its overall properties and make it as suitable for energy storage applications as other well-studied polymers in this substance class, such as polyaniline and polypyrrole. In this study, by synthesizing Pbp in surfactant-supported acidic medium, we were able to show that the physicochemical and electrochemical properties of Pbp-based electrodes are strongly influenced by the respective polymerization conditions. Through appropriate optimization of various reaction parameters, a significant enhancement of the thermal stability (up to 549.9 °C) and the electrochemical properties could be achieved. A maximum specific capacitance of 166.0 ± 2.0 F g^−1^ with an excellent cycle stability of 87% after 5000 cycles at a current density of 1 A g^−1^ was achieved. In addition, a particularly high-power density of 2.75 kW kg^−1^ was obtained for this polybenzopyrrole, having a gravimetric energy density of 17 Wh kg^−1^. The results show that polybenzopyrroles are suitable candidates to compete with other conducting polymers as electrode materials for next-generation Faradaic supercapacitors. In addition, the results of the current study can also be easily applied to other systems and used for adaptations or new syntheses of advanced hybrid/composite Pbp-based electrode materials.

## 1. Introduction

With the emergence of energy based unconventional digital technologies, and expeditious expansion in population and industries across the globe, substantial environmental and energy supply/demand issues have been witnessed in the previous decades [1,2,3]. Progress in the development of renewable energy conversion and storage technologies is, therefore, one of the most urgent societal challenges of our time [4,5]. In this regard, supercapacitors, as viable alternatives, have opened the way to large scale electrochemical energy storage for later use. They offer numerous advantages such as cost-effectiveness, non-toxicity, moderate energy density, high-power density, and faster charging/discharging [1,3,5,6,7]. Supercapacitors find significant applications in aviation, hybrid vehicles, voltage stabilizers, electronics, and railway transport [8].

Faradaic supercapacitors, exhibiting pseudocapacitance, utilize redox-active electrode materials such as metal oxides and conducting polymers to store charge through Faradaic processes (redox reactions) at the electrode surface [7,8]. Polybenzopyrrole (Pbp, (C_8_H_6_N^+^X^-^)_n_), a nitrogen containing redox-active conjugated polymer obtained by the oxidative polymerization of benzopyrrole (C_8_H_7_N), is regarded as a new electrode material for Faradaic supercapacitors. Although Pbp was described as a low-cost electrode material with significant redox activity, sufficient thermal stability, and a reasonably slow degradation rate compared to polypyrrole and poly(para-phenylene), it has an unsatisfactorily low specific capacitance. In addition, Pbp as a redox-active electrode material is usually disregarded due to its insufficient surface area, difficulty to attain a desirable morphology, low electrical conductivity, and costly electrode fabrication. Volume contraction and expansion restrict applications of Pbp by forfeiting its electrochemical stability, which is a key problem in the fabrication of supercapacitors. Hereafter, challenges to be overcome include improving processability, energy holding capacitance, power density, lowering cost, and reducing environmental impact by reducing the overuse of harmful chemicals [9,10,11,12,13].

To achieve a performance exceeding existing limits, especially in terms of energy density and durability, researchers have attempted to develop and tune the specific capacitance of Pbp through synthesis using numerous strategies, such as composite fabrication, modification with other materials in blends, and electrode fabrication methods [7,10,14]. In this regard, few studies on the Pbp and its composites for supercapacitors have been reported. Although Pbp and Pbp composite materials appear to be appropriate for Faradaic supercapacitor applications, there are some drawbacks which limit their use: insolubility, low yield, high cost (due to the use of expensive organic carbonaceous and/or inorganic metal oxides as compositing component), and high sensitivity of unstable synthesis conditions. In addition, the obtained product requires complex postprocessing, including the addition of a costly and often toxic binder for the electrode production of the Pbp composite, making the product uneconomical. However, improving the supercapacitive and electrical properties of Pbp to meet the requirements of Faradaic supercapacitors is still desirable. Therefore, one must emphasize the inherent properties of Pbp first, after which a comprehensive optimization of the full Faradaic supercapacitor should be performed under practically relevant conditions [7].

Traditional chemical oxidative polymerization in the presence of acidic dopants and oxidant is a simple, easy, template free pathway to obtain conjugated polymer. This method can be performed to obtain large scale and high-quality products. It is known that the polymerization yield, physicochemical, and electrochemical properties of the synthesized materials are greatly affected by the synthesis process, as well as by the nature of the oxidizing agent and dopant anion incorporated into its backbone. The oxidizing agent initiates the polymerization by removing an electron from the monomer to generate monomer radical cations, which combines with additional benzopyrroles in a deprotonation/oxidation cascade to form the polymer (Scholl reaction). On the other hand, dopants such as hydrochloric acid (HCl) and perchloric acid (HClO_4_) allow the protonation of nitrogen atoms in the polymer to generate charge carriers (polarons/bipolarons) that move along the Pbp backbone through a hopping mechanism and, consequently, result in an intrinsically conducting material. Common surfactants such as sodium dodecyl sulfate (SDS), cetyltrimethylammonium bromide (CTAB), and tween 80 have also been integrated with the protonic acid to improve optical, electrical, and structural characteristics of these materials. The amount of dopant and distribution of its counter ions along the main chain effects the polymerization yield, mobility of charge carriers, and structural properties. To this end, keeping in mind all this information, we opted to synthesize Pbp as a suitable and appropriate material for energy storage by having the right choice of oxidant, dopants, and controlling oxidant/monomer, as well as dopants mole ratio [7,11,15,16]. Although extensive literature is available on the effect of various synthesis parameters on the properties of other conducting polymers such as polyaniline, polypyrrole, poly(o-toluidine), etc., to the best of our knowledge, no single report in literature compared the effects of all reaction parameters on the physical, chemical, and, more particularly, on the electrochemical characteristics of doped Pbp-based materials. Thus, deep understanding of the formation mechanism and performance of Pbp is significantly important for aqueous supercapacitors that motivated us to prepare Pbp with consistent quality and high electrochemical performance.

Hence, in the current assessment, we present a systematic screening approach aiming to explore the effect of oxidant/monomer mole ratio and concentration of dopants (DBSA and H_2_SO_4_) on the structural, morphological, and electrochemical characteristics of the resulted Pbp-based electrode materials. The overall outcomes disclosed that all the aforesaid reaction conditions considerably impacted the structural properties of Pbp at different levels. This, in turn, influenced its potential towards aqueous redox supercapacitors in acidic medium. Thus, the optimization of synthesis parameters made it possible to obtain Pbp electrode material with a moderately high energy density and rate capability. The results convincingly showed that Pbp obtained in the present work could be a competitive electrode material in the race of Faradaic supercapacitors electrodes.

## 2. Materials and Methods

### 2.1. Chemicals

Benzopyrrole (C_8_H_7_N, >99%), dodecylbenzenesulfonic acid (DBSA, >95%), ammonium persulfate (APS, >98%), acetone (99%), and chloroform (>99.8%) were purchased from Sigma Aldrich (St. Louis, MI, USA). Toluene and sulfuric acid (H_2_SO_4_, 98%) were obtained from Scharlau (Barcelona, CAT, Spain). 2-Propanol and dimethyl sulfoxide (DMSO) were purchased from Daejung (Seoul, Korea). All the chemicals were of analytical grade and were used as received. Millipore deionized water (>18 MΩ) was used throughout the experimental work.

### 2.2. Synthesis Procedure of Polybenzopyrrole (Pbp)

**Scheme 1:**
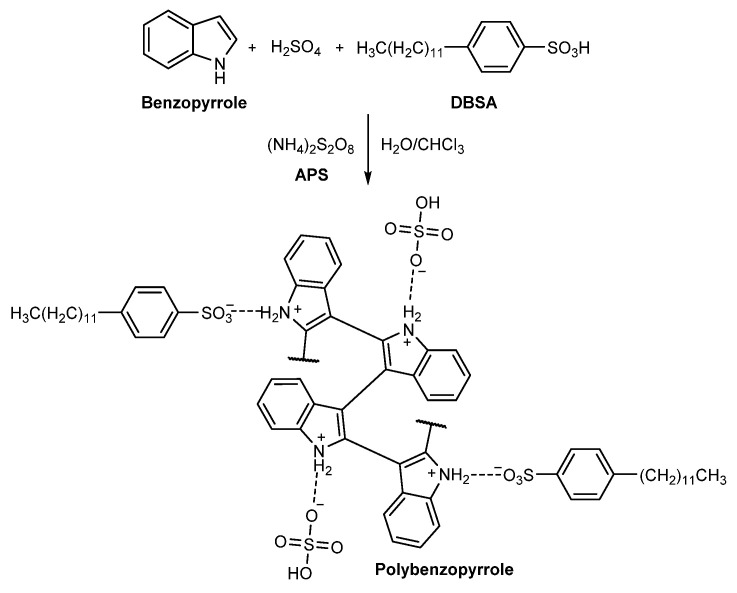
Schematic synthesis of Pbp in H_2_SO_4_/DBSA.

The synthesis of Pbp was executed via the anionic surfactant assisted polymerization method (Scheme 1). In a 250 mL flask, 40 mL of freshly prepared 0.1 M H_2_SO_4_ was added, followed by addition of DBSA (1.0–2.0 mL) and APS (7.30 mmol) under rapid stirring (800 rpm) for 5–10 min. The polymerization was initiated by steady addition (1 mL/min) of the required amount of benzopyrrole (APS:BP 0.25–3.0) in chloroform. Directly after the first addition of the monomer, the solution turned slightly pink indicating the start of the polymerization. The mixture was stirred constantly for 24 h at room temperature. In the course of time, the mixture turned dark green. Addition of a mixture of acetone:water 1:1 (*v*/*v*) precipitated the dark green doped Pbp. The precipitate was filtered and thoroughly washed with acetone until the filtrate became colorless. The polymer was dried at 60 °C in vacuum for 24 h before its analysis.

A series of experiments were conducted in which reaction parameters, such as the molar ratio of APS to benzopyrrole (APS/BP), the concentration of H_2_SO_4_, and the amount of DBSA, were varied to determine the optimal reaction conditions for the synthesis of Pbp. In a first set of experiments, the molar ratio of APS/BS was changed while the amounts of H_2_SO_4_ and DBSA were kept constant (Table 1, entries 1–7). Then, the H_2_SO_4_ concentration was varied (entries 9–12) and the amount of DBSA was changed (entries 13–16), while all other parameters remained unchanged.

### 2.3. Analysis and Characterization

The percentage yield was selected as a benchmark for optimization of Pbp. Equation (1) is used to calculate the yield of all Pbp [17].

Percent yield = (weight of polymer/weight of monomer) × 100(1)

To ascertain structural implications of each sample, a Fourier Transform Infrared (FTIR, Affinity-1S) spectrometer from Shimadzu (Kyoto, Japan) was used to record spectra of each Pbp powder sample in the range of 450 to 4000 cm^−1^. Scanning Electron Microscope (SEM) (Supra 55VP, ZEISS FEGSEM, Jena, Germany) was used to analyze morphology of the samples. Moreover, SEM-Energy Dispersive X-ray (SEM-EDX, Oxford Instrument, Abingdon, UK) analysis was employed for the elemental analysis of polymer samples. X-ray diffraction (XRD) patterns were acquired using a Bruker D8 Advance diffractometer with Cu Kα radiation (λ = 1.54 Å) generated by accelerating electrons over 40 kV at an anode current of 35 mA. The intensity of scattered X-rays was measured in a 2θ-range of 10–50°, step width of 0.0164°, 1 step/s. The optical absorption spectroscopy of all samples was measured with an Ultraviolet-Visible spectrophotometer from PerkinElmer (Waltham, MA, USA) in the wavelength range of 200–1000 nm. The polymers were dispersed in dimethyl sulfoxide and a quartz cuvette (1 cm path length) was employed for measurements. A PerkinElmer Thermogravimetric Analyzer (TGA, Waltham, MA, USA) was employed to observe thermal properties of the samples by heating over a range of 20 to 800 °C at the rate of 10 °C per minute under nitrogen (N_2_) environment. Approximately 5 mg of each Pbp sample was used to investigate the change in polymer weight as a function of temperature.

### 2.4. Electrochemical Characterization

The electrochemical properties of redox-responsive Pbp electrodes were firstly evaluated in a three-electrode setup on a Reference 3000 ZRA Potentiostate/Galvanostate from Gamry Instruments (Warminster, PA, USA). The working electrode was prepared through depositing 0.2 mg Pbp on a gold sheet electrode (1 × 1 cm^2^). A dispersion of Pbp was prepared in 1:2 mixture of 2-propanol and toluene, which was then applied over the electrode and left to dry at room temperate for about 10 min. Another gold sheet and a silver/silver chloride (Ag/AgCl, KCl_sat_ in H_2_O) electrode were used as the counter electrode and reference electrode, respectively. The electrochemical performance of polymer electrodes was evaluated in 1 M H_2_SO_4_ through Cyclic Voltammetry (CV), Galvanostatic charge-discharge (GCD), and Electrochemical Impedance Spectroscopy (EIS). CV of gold modified Pbp electrodes were recorded at different scan rates from 20 to 100 mV s^−1^ in a potential window between −0.4 to 1.1 V. GCD was conducted at various current densities ranging from 0.35 to 5.0 A g^−1^ to calculate gravimetric specific capacitance of the polymer electrodes. In order to calculate gravimetric specific capacitance of the active electrode material from CV and GCD, the following Equations (2) and (3) were used [6,18]:

*C_s_* = ∫*I dv*/(2 × *m* × *s* × ∆*V*) (2)

*C_s_* = (*I* × ∆*t*)/(∆*V* × *m*) (3)

where *C_s_* is the specific capacitance (F g^−1^), *∫I dv* is integrated area of CV (C), s is the potential sweep rate (mV s^−1^), *I* is the current (A), ∆*t* is the discharge time (s), ∆*V* is the sweep potential window (V), and *m* is the mass of active electrode material (g). In addition, potentiostatic EIS was employed in the frequency range of 100 kHz to 40 mHz at *E*_DC_ = 200 mV to evaluate solution resistance, charge transfer resistance, and equivalent series resistance of the Pbp electrode in three-electrode system.

Moreover, the actual electrochemical performance of the Pbp was studied in a symmetric two-electrode configuration. The supercapacitor cell was assembled by placing two Pbp coated gold sheet electrodes in 1 M H_2_SO_4_ with a unit distance apart. CVs were obtained at 20–100 mV s^−1^ potential scan rates, whereas GCD curves were recorded at 0.5 to 5.0 A g^−1^. From GCD, the gravimetric specific capacitance and coulomb efficiency (*η_t_*) in two-electrode setup were calculated by Equations (4) and (5), respectively [18,19].

*C_s_* = 4(*I* × ∆*t*)/(∆*V* × *m*) (4)

*η_t_* = (*t_d_*/*t_c_*) × 100 (5)

where *V_d_* is the voltage drop (V), *t_d_* is discharge time (s), and *t_c_* is charging time (s). The Ragone plot is obtained by calculating the gravimetric energy density and power capability using Equations (6) and (7), respectively, where *E* is the gravimetric energy density (Wh kg^−1^), P is the gravimetric power density (W kg^−1^), *C_s_* is the specific capacitance, ∆*V* is the operating potential window, and ∆*t* is discharge time [18].

*E* = (1/8) *C_s_*∆*V*^2^ (6)

*P* = *E*/∆*t* (7)

The electrochemical cycling stability of the Pbp electrode was examined by employing GCD at 1 A g^−1^ for 5000 cycles in symmetric two-electrode system.

In order to evaluate the internal resistance, ion diffusion, and charge transfer characteristics, the assembled symmetric two-electrode device is subjected to EIS in the frequency range of 100 kHz to 40 mHz, while keeping potential constant at *E_DC_* = 200 mV.

## 3. Results and Discussion

### 3.1. Optimisation of the Synthesis Conditions

The polymerization yield and the overall properties, especially the electrochemical activity of the synthesized polymers, is strongly influenced by the reaction parameters (APS/BP molar ratio, concentration of H_2_SO_4_, and amount of DBSA). In order to obtain optimal conditions for the best physical, chemical, and electrochemical properties along with high yield, the abovementioned variables were investigated systematically (Figure S1). The results show that a maximum yield of 56% is obtained at synthesis conditions using an APS/BP ratio of 2.5 in combination with 0.1 M H_2_SO_4_ and 1 mL DBSA. This is due to the formation of gradual, elongated, and regular Pbp chains that reach their optimized form under the prevailing reaction conditions. Pbp polymerize stepwise into the macromolecules and thus the degree of polymerization increases steadily. Deviating from the reaction conditions, either an unselective reaction occurred, or the conversion was too low [7,17,18,19,20,21,22]. The results of this study show good agreement with the previous studies reported for other similar conducting polymers [17,18,19,20,21,22].

Moreover, the variation of the DBSA amount seems to have a minor effect on the yield of the polymer compared to that of H_2_SO_4_ and APS. Based on the observed results, it is expected that both dopants essentially interacted with the polymer chain. The incorporation of the dopants into the Pbp backbone was validated by the structural, optical, and morphological studies. Besides, the polymer samples S-0.25A/B, S-1.0A/B, S-3.0A/B, and S-2.0H were not investigated further due to insufficient polymerization yield.

### 3.2. FTIR Spectroscopy

**Figure 1:**
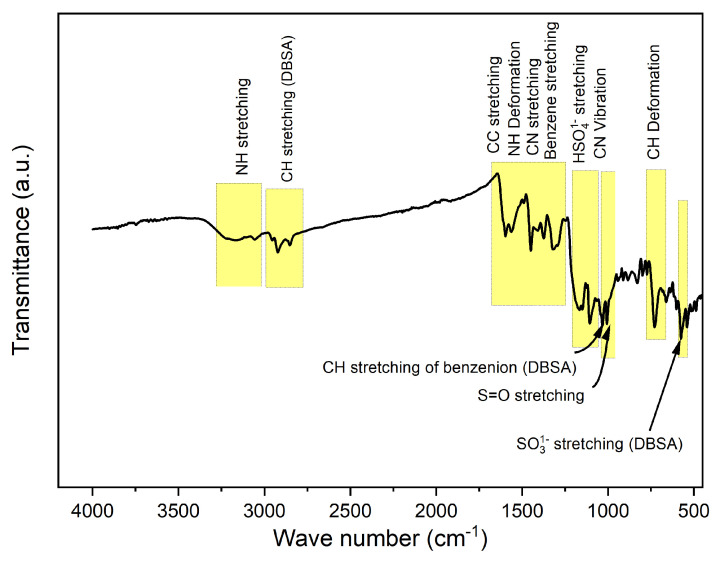
Representative FTIR spectrum of Pbp S-2.5A/B doped with H_2_SO_4_ and DBSA.

To confirm the success of the synthesis, the samples were first analyzed by FTIR spectroscopy for the presence of representative functional groups as a function of the different reaction parameters. Figure 1 shows a typical FTIR spectrum of Pbp S-2.5A/B. The absorption band of the N-H stretching at 3167 cm^−1^ and the deformation vibrations of the N-H bond at 1559 cm^−1^ are characteristic. The presence of the N-H bond proves that the nitrogen species does not form a chemical bond during polymerization. Consequently, the polymerization must occur at C2 and C3 position of the benzopyrrole, which are known to be the most reactive in oxidative reactions. The most probable structure of Pbp is shown in Scheme 1 [15,16]. The appearance of the band at 728 cm^−1^ is the signature of the out-of-plane deformation of the C-H bond of the benzene ring. This also proves that the benzene ring does not participate in the polymerization reaction. Moreover, the bands at 1447 and 1106 cm^−1^ show the corresponding stretching vibration of the C-N bond induced by the doping [23]. Furthermore, the typical vibration of the C-C polymer chain could be identified by the characteristic peak at 1599 cm^−1^ [24]. The incorporation of DBSA into the Pbp carbon skeleton could be confirmed from the band of the symmetric stretching vibration of S=O, SO_3_^−^, and C-H stretching of the benzene ring of DBSA, at 1006, 576, and 1030 cm^−1^, respectively. The C-H stretching of alkyl chain of DBSA is evidenced by the doublet at 2920 and 2850 cm^−1^ [17,22,25]. Besides, the peak situated at 1168 cm^−1^ is ascribed to the stretching of HSO_4_^−^ anion of H_2_SO_4_ showing its successful incorporation into Pbp backbone [21].

The FTIR spectra of all Pbp samples with varying reaction parameters are presented in Figure S2. Upon altering the reaction conditions, all the Pbp polymers exhibited nearly the same IR vibration spectra with little irregularity in peak intensities and positions. Henceforth, FTIR spectra signify the degree of conjugation and sensitivity of structural characteristics of developed well-doped Pbp samples to the various reaction parameters. Our results were in good agreement with the literature and also coincide with our percent yield study [16,26,27,28].

### 3.3. Morphological Analysis

**Figure 2:**
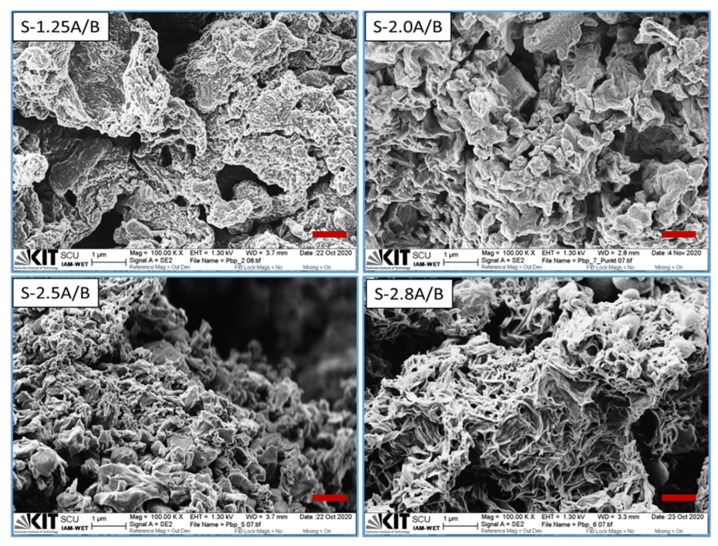
SEM of Pbp samples with various APS/BP = 1.25 to 2.8 mole ratio (0.1 M H_2_SO_4_, 1.0 mL DBSA). The red bars in the lower left half of each image correspond to 1 μm.

The morphological characteristics of all Pbp samples as-prepared under different APS/BP molar ratios are shown in Figure 2. The SEM image of as-synthesized S-1.25A/B shows the appearance of a spongy, coral-like morphology. When the APS/BP molar ratio is increased, clustered microparticles with irregular shape and different sizes appear in the polymer samples S-2.0A/B and S-2.5A/B. The agglomeration and crosslinking of the particles, which form a non-uniform, clump-like morphology, make it difficult to identify the shape and size of the microparticles. However, the agglomeration is more pronounced in S-2.0A/B compared to S-2.5A/B. Numerous gaps of different sizes can be seen in the microstructure. For S-2.5A/B, the surface of the larger agglomerates is covered with smaller nanosized particles with a broad distribution ~80–350 nm. Towards a higher APS/BP molar ratio (2.8), interconnected, fused, and short nanostructures (with an average diameter of 40–100 nm) appeared in S-2.8A/B together with irregular agglomerates. Thus, the morphology showed a great dependence on the APS concentration. Such morphological changes with changes in the amount of APS have also previously been reported for other polymers [7,29]. Although Pbp prepared with higher APS/BP molar ratio exhibited more regular nanostructured morphology, the high degree of overoxidation confirmed by FTIR cannot be neglected since it is known to have negative effects on the electrochemical activity (see Section 3.7).

**Figure 3:**
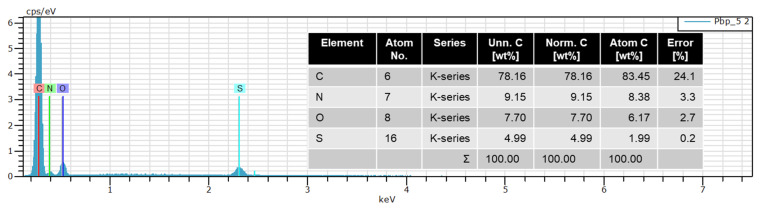
EDX analysis of S-2.5A/B.

The SEM images of the Pbp samples with different concentrations of H_2_SO_4_ and DBSA are shown in Figures S3 and S4, respectively. Interestingly, the morphology of the Pbp changed from a spongy to compact appearance when the concentration of H_2_SO_4_ and the amount of DBSA were changed. Moreover, the microparticles showed variations in size and extinction of agglomeration of the particles. This could be due to the formation of polarons/bipolarons producing large particles and micelle fusion of the surfactant due to the varying amount of DBSA. Studies that have reported the effect of acid concentration and amount of surfactant on the morphology of the conjugated polymers also found a similar effect [15,25,29,30]. Figure 3 shows EDX analysis of the representative Pbp sample (S-2.5A/B). The presence of C, N, O, and S elements in EDX spectra confirms the successful incorporation of DBSA and H_2_SO_4_ in the Pbp backbone. The sulfur content varies with the stepwise variation in concentration of both H_2_SO_4_ and DBSA dopants during the synthesis. This is an indication of incorporation of sulfur from both the dopants. In addition, Pbp was prepared separately in aqueous medium using H_2_SO_4_ (without DBSA) and DBSA (without H_2_SO_4_) using 2.5 A/B mole ratio. The sulfur content due to H_2_SO_4_ and DBSA was found to be 3.69 and 4.14%, respectively. The value of sulfur content increased to 4.99% in the case of Pbp prepared in DBSA assisted aqueous H_2_SO_4_ medium. Moreover, the increment in carbon content from 72.02% for Pbp prepared with H_2_SO_4_ only to 78.16% in Pbp prepared with both H_2_SO_4_ and DBSA is ascribable to the incorporation of long carbon chain of DBSA. This indicates that both DBSA and H_2_SO_4_ dopants are incorporated in the Pbp. A similar study on synthesis of conjugated polymer with H_2_SO_4_ and sodium lauryl sulfate has been reported previously [31].

### 3.4. XRD Analysis

**Figure 4:**
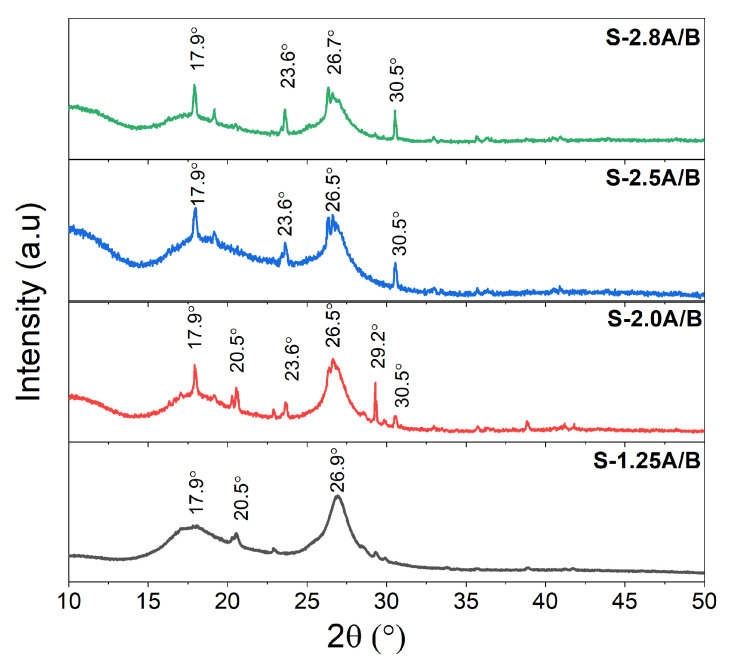
XRD of Pbp samples with different APS/BP molar ratios during synthesis between 1.25 to 2.8 (0.1 M H_2_SO_4_, 1.0 mL DBSA).

XRD analysis was employed in order to evaluate the effect of various reaction conditions on the crystallinity of the polymer. The polymer crystallinity is a significant factor for its electrochemical activity. The representative diffractograms of Pbp are depicted in Figure 4. The broadened and sharp reflection peaks characteristic of Pbp, between 2θ~17.9 and 26.9°, can be found in all samples. The occurrence of a broadened and a sharp peak indicate semicrystalline polybenzopyrrole, with the broadened feature representing the amorphous phase while the occurrence of the sharp peaks represents the crystalline phase of Pbp [32,33,34]. Other small peaks observed at 2θ~20.5, 23.6, 29.2, and 30.5° were thought to be induced by the alkyl chain branches of DBSA anion integrated in the Pbp backbone. These peaks appeared at various 2θ due to the orientation of DBSA chains in different directions in the Pbp chain [25].

At a lower APS/BP molar ratio (1.25), two peaks are clearly seen at 2θ = 17.9 and 26.9°. The intensity of the characteristic peaks seems to increase with the increase of the APS/BP molar ratio up to 2.8. With the variation of the reaction parameters, the increase in peak height along with the appearance of additional peaks indicates the achievement of a more compact and regular arrangement of the molecules, which may have led to better electron transfer and intermolecular hopping of charge carriers. Another reason may be the production of longer conjugated polymer chains with the incorporation of DBSA counter anions per monomer unit. Previous literature has also noted the increase in crystallinity of Pbp-based polymers, and others to some extent, with the change in reaction conditions [35]. In contrast to the observations from FTIR (Figure S2a,b), no increase in the degree of oxidation was observed from the XRD patterns with the increase in the APS/BP molar ratio (up to 2.8).

XRD patterns of Pbp polymers with varying H_2_SO_4_ and DBSA amounts, as presented in Figures S5 and S6, also show a signature broad hump and a sharp peak along with extra short peaks at various 2θ values. With the change in reaction parameters, 2θ values and peak intensity changed. This implies to the occurrence of structural changes which resulted in variation in the crystallinity, order, and length of conjugated polymer chains. A similar effect of variation in acid dopant and surfactant on the other conjugated polymers, polypyrrole, and polyaniline was also observed by other works [25,26,29].

### 3.5. Photochemical Properties of the Polybenzopyrrols

The photochemical analysis of the polybenzopyrroles was performed to obtain additional information about the charge distribution of the conductive polymer chains. The UV-Vis absorption spectra recorded for all Pbp polymers are shown in Figure S7. All spectra show the highest absorption bands in the lower wavelength range of 250–400 nm. The first (ca. 273 nm) and the second (ca. 311 nm) absorption bands are assigned to the π-π* and n-π* transitions running from the valence to the conduction band in the benzoid rings of the conjugated Pbp. All spectra showed a third peak near 350 nm and broadened features at 378, 397, and 740 nm. The presence of these bands are the characteristics of such conjugated polymers originating from the broadening of conjugation and excitation from the valence band to the bond polaron state of doped Pbp [36,37,38].

Slight shifts in the bands are observed with the change in reaction parameters. These can be attributed to increased or decreased delocalization of charge along the polymer chain. It is assumed that with the increase in delocalization, the required excitation energy decreases and the wavelength shifts bathochrome. Similarly, blue shifts occurred with the decrease in delocalization, which can be attributed to the splitting of the polymer chains into shorter chain fragments. From these spectral results, it is concluded that longer conjugated chains and broader molecular distribution of Pbp polymers are obtained under the optimized reaction conditions [36]. Similar effects of the reaction parameters on the absorption spectra of conjugated polymers have already been described in the literature [26,39,40].

### 3.6. Thermogravimetric Analysis

**Figure 5:**
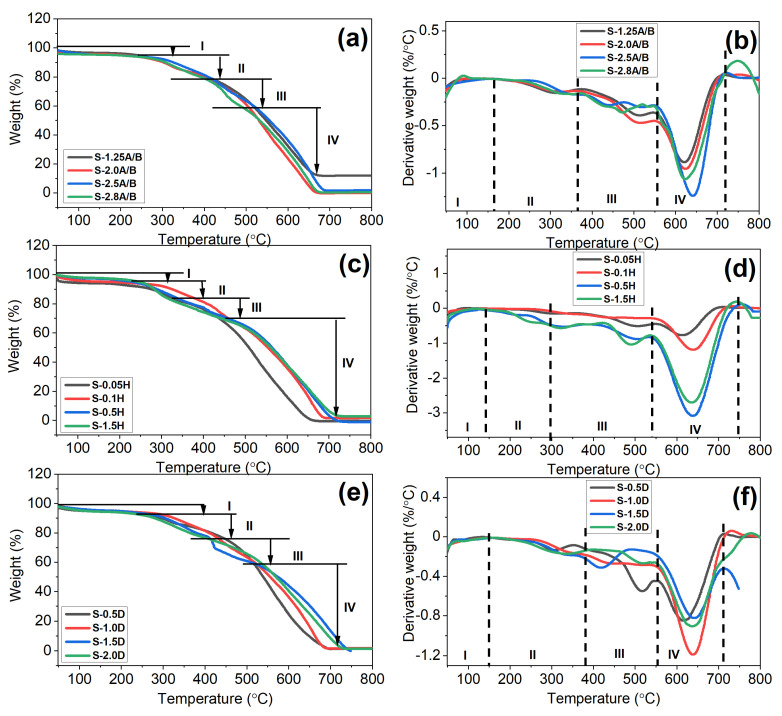
TGA and DTGA profiles of as-synthesized Pbp materials at different (**a**,**b**) APS/BP mole ratio (1.25–2.8) through varying benzopyrrole concentration, (**c**,**d**) H_2_SO_4_ concentration (0.05–1.5 M), and (**e**,**f**) DBSA concentration (0.5–2.0 mL).

The resistance of polymers at high temperatures is important for their application in advanced technologies. At high temperatures, there is a loss of electrical conductivity due to the elimination of dopants from the polymer. This limits the use of polymers in sensors, batteries, supercapacitors, electromagnetic shielding, etc. [21]. Thermal stability is one of the competing properties of Pbp compared to the other conjugated polymers. Therefore, we used TGA to study the thermal stability of Pbp powder samples by monitoring the reduction in mass as a function of temperature. The TGA profiles of all Pbp samples are shown in Figure 5. The TGA/DTGA curves of each polymer sample exhibited a common feature, a four-step thermal decomposition. An initial weight loss (~6%) at a temperature >150 °C is associated with the evaporation of entrapped water, solvent, or monomer molecules in the hygroscopic polymer matrix. From then on, there is a second weight loss in the range of 160 to 463 °C due to the elimination of the oxidant and a third mass loss due to the removal of dopants is observed at 360–550 °C for various samples. Finally, the backbone of the Pbp chains breaks at a temperature of >550 °C and shows a significant weight loss.

TGA/DTGA curves of Pbp polymers with different reaction parameters showed variations in the rate and temperature of degradation (Table S1). This indicates a well-developed interaction between dopant and Pbp leading to variable acquired stability of the polymer matrix [10,15,41]. This is due to the broad molecular weight distribution of Pbp synthesized under the prevailing conditions of DBSA and H_2_SO_4_, which leads to better interaction with the polymer chain. The results of our work show that Pbp synthesized with an APS/BP molar ratio of 2.5, 0.1 M H_2_SO_4_ and 1.0 mL DBSA, represents the optimum that achieves better thermal stability (549.9 °C), which is comparatively higher than those reported in the literature [10,12,15]. It is suspected that the synthesized Pbp has great significance in applications operating at elevated temperatures.

In summary, the best compromise between reaction parameters and material properties was achieved for the sample prepared by the synthesis procedure with an APS/BP molar ratio of 2.5, with 0.1 M H_2_SO_4_ and 1.0 mL DBSA. To determine whether or not the finding for the application of the Pbp polymers in aqueous redox supercapacitors (see Section 3.7) could be valid, their electrochemical performance was evaluated using electroanalytical techniques.

### 3.7. Three-Electrode Cell Electrochemical Performance

#### 3.7.1. Cyclic Voltammetry

Exploring the electrochemical performance of Pbp electrode materials is obligatory in order to expand its practical implementation in aqueous redox supercapacitors. To this end, Cyclic Voltammetry (CV) was performed in a three-electrode configuration using 1 M H_2_SO_4_ electrolyte solution. As mentioned earlier, the electrochemical charge storage is the significant parameter to evaluate a supercapacitor’s performance. Thus, area under CV plots was integrated by using Gamry Echem Analyst software to obtain the electrochemical charge stored by each electrode (Table S2).

**Figure 6:**
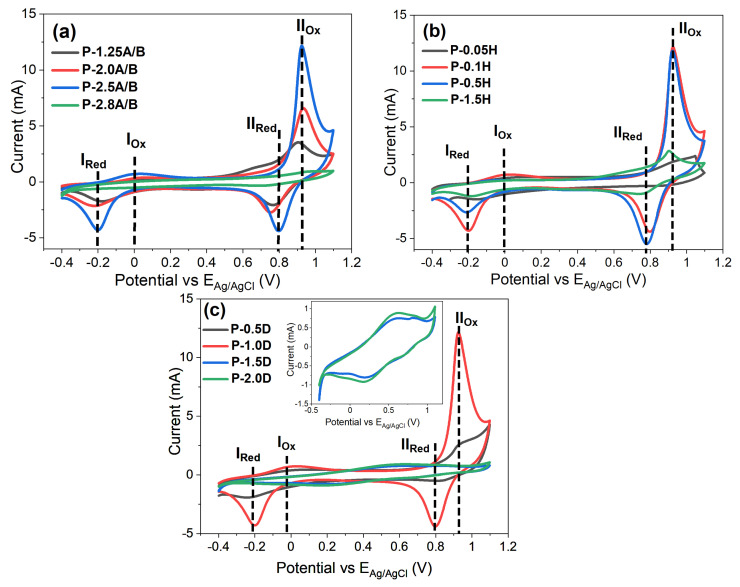
CV plots of Pbp coated gold electrodes from (**a**) P-1.25A/B to P-2.8A/B, (**b**) P-0.05H to P-1.5H, (**c**) P-0.5D to P-2.0D at 100 mV s^−1^ in 1 M H_2_SO_4_. Inset: CV of P-1.5D (blue) and P-2.0D (green).

The variation in CVs with the change in APS/BP mole ratio, H_2_SO_4_ concentration, and DBSA amount can be seen in Figure 6. The potential window was kept between −0.4 and 1.1 V. All the CV plots exhibited pseudocapacitive nature, which can be clearly seen from the appearance of a sharp and quasi-reversible pair of characteristic oxidation-reduction couple (I_Ox_/I_Red_ and II_Ox_/II_Red_) with alteration in their positions for various Pbp samples obtained under various reaction conditions which exhibit structural variations. This might be ascribed to the well-developed interaction between dopant’s counter ions and Pbp. Cyclic voltammograms with such redox couples are considered to form a stable cationic state of Pbp from a delocalized polar state by charge carrier conversion [7,32].

In acidic electrolyte, the first redox couple is associated with a single electron transfer of the polymer chain. The second redox pair, on the other hand, corresponds to a proton-coupled electron transfer process which is triggered by protonation and deprotonation during doping (introduction of the counter anion from the electrolyte into the polymer chain) and de-doping (expulsion of the counter anion from the electrolyte out of the polymer chain). Pbp has been proven (by FTIR) to be doped with bulky H_3_C(CH_2_)_11_(C_6_H_6_)SO_3_^−^ anions (from DBSA) and a small amount of HSO4^-^ anions (from H_2_SO_4_). It can be expected that the H^+^ and SO_4_^2−^ ions from the electrolyte interact with the counter ions of the dopants in the polymer chain during the electrochemical oxidation-reduction processes. Electrolytic ions can displace and substitute the dopants incorporated in the polymer chain, H_3_C(CH_2_)_11_(C_6_H_6_)SO_3_^−^ and HSO_4_^−^, during the redox process. Since the hydrogen sulfate and sulfate ions have a greater mobility than the sterically hindered DBSA anions, an exchange of these is preferred. Thus, it can be concluded that the DBSA anions fixed in the polymer structure contribute to high electrochemical stability, while the sulfate ions enable reversible doping [42,43].

The peak potentials and charge storage values varied under different conditions of component concentrations. The reduced peak intensities of some polymer samples under the described conditions resulted in their uncertain position and width. This can be interpreted as increased irreversibility and an internal resistance drop in these electrodes, leading to their poor electrochemical performance. Thus, the change in peak intensities and symmetry signifies whether the Pbp sample retains its electroactivity or not [7,30,32,42].

To verify these results, we calculated the specific capacitance of each sample at different scan rates using Equation (2). The specific capacitance of all samples at different scan rates is shown in Figure S8c–e. The highest specific capacitance of 774 F g^−1^ was obtained at 20 mV s^−1^. However, the decrease in specific capacitance with the increase in scan rate (20–100 mV s^−1^) is observed for all samples. The occurrence of this phenomenon can be attributed to internal resistance or diffusion limitations with sequential increase in scan rate. In other words, the limited penetration of ions into the inner surface of the electrodes does not allow the electrolyte ions to occupy all available active sites. This leads to insufficient redox processes, consequently the charge storage and specific capacitance is reduced [42,44,45].

The appearance of obvious and well-resolved redox peaks and larger than average confined area in the CV plots for P-2.5A/B, P-0.1H, and P-1.0D electrodes implies a high electrochemical charge storage of 38.95 mC and a high specific capacitance of 774 F g^−1^ (20 mV s^−1^) compared to other Pbp electrodes. This behavior is underpinned by performance against aqueous supercapacitors, so achieving the best conditions for desirable properties was validated for P-2.5A/B, P-0.1H, and P-1.0D electrodes. Since these samples were obtained under the same reaction parameters, we named them Pbp@HD. We selected the sample for further detailed electrochemical studies for aqueous supercapacitor application [44]. The energy storage mechanism in supercapacitors can be surface-controlled or diffusion-controlled. In the following, the peak current density from CV plots at different scan rates is used to confirm the energy storage mechanism in Pbp electrodes using the equation (*I* = *a s^b^* or *log I* = *log a* + *b log s*), where *I* is the peak current, *s* is the scan rate, and *a* and *b* are arbitrary constants. Here, *b* determines the energy storage mechanism. If its value is 0.5, the process is diffusion controlled, while if its value is 1.0, the charge storage mechanism is a surface-controlled process. The value of b can be determined from the slope of the curve between log s and log I (Figure S8b). Interestingly, the value of b for the peak anodic and cathodic currents for the Pbp@HD electrode is 0.83 and 0.85, respectively, suggesting that charge storage occurs through both capacitive and intercalation of ions by diffusion into the electrode material [5,6].

#### 3.7.2. Galvanostatic Charge-Discharge

**Figure 7:**
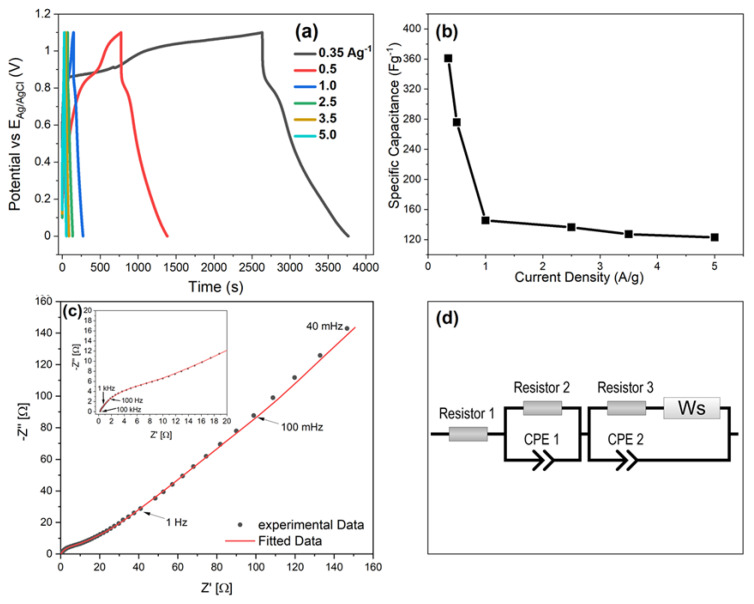
(**a**) Galvanostatic charge-discharge plots and (**b**) specific capacitance as a function of current density, and (**c**) EIS; Inset: EIS of Pbp@HD between 100 kHz and 40 mHz, and (**d**) electrochemical equivalent circuit (CPE = constant phase element, Ws = short Warburg Diffusion).

The Pbp@HD electrodes were subjected to charge-discharge measurements in the pre-evaluated potential window (0 to 1.1 V) at various current densities from 0.35 to 5.0 A g^−1^. As can be seen in Figure 7a, the GCD curves have a distorted triangular shape with the foremost deviation representing the pseudocapacitive behavior of all Pbp electrodes. As the current density increases, a shortening of the charge-discharge time is observed. Moreover, the highest gravimetric specific capacitance of 361 ± 1.2 F g^−1^, calculated according to Equation (3), is obtained for Pbp@HD at 0.35 A g^−1^. The significantly high specific capacitance could only be explained by the desirable structural properties of the polymer, which provides a porous network leading to a relatively long discharge time. With increasing current density, the specific capacitance decreased to 276 ± 0.96 F g^−1^ (0.5 A g^−1^), 145.5 ± 1.2 F g^−1^ (1.0 A g^−1^), 136 ± 0.6 F g^−1^ (2.5 A g^−1^), 127.3 ± 2.4 F g^−1^ (3.5 A g^−1^), and 122.7 ± 1.6 F g^−1^ (5.0 A g^−1).^ for Pbp@HD (Figure 7b). This is due to diffusion limitations by ions diffusing into the inner surfaces of the electrode material at higher current densities [31]. Thus, the Pbp@HD electrode showed a relatively higher specific capacitance of 774 F g^−1^ (20 mV s^−1^) and 361 ± 1.2 F g^−1^ (0.35 A g^−1^) from CV and GCD, which is much better compared to pristine Pbp (120 F g^−1^) reported earlier [46].

#### 3.7.3. Electrochemical Impedance Spectroscopy

EIS was used to further study the internal resistance, charge transfer kinetics, and ion diffusion processes of Pbp@HD. Electrochemical impedance spectroscopy can be used to exploit the different time constants of these processes to separate them and their respective contributions to the total loss. Figure 7c shows the Nyquist plot of the measured Pbp@HD. The frequency range was between 100 kHz to 40 mHz, using a perturbation amplitude of 10 mV. The recorded spectra can be divided into three areas: (1) the intersection with the x-axis (>100 kHz) contains the charge transfer resistance from current collector, wiring, electrical, and ionic transport through electrode and electrolyte, (2) the implied semicircle in the mid-frequency range (100 kHz–~30 Hz) contributing to the charge transfer resistance of the polymer, and (3) a monotonically increasing straight line at frequencies below ~30 Hz typically associated with slow diffusion processes, leading to a more idealized capacitive behavior.

For the analysis of the individual contributions, a simple electrical equivalent circuit diagram was used to describe four electrochemical processes: (i) the series resistance (resistor 1) caused by a mixed conduction of ion species and electric resistance and the electrolyte solution resistance, (ii) the charge transfer resistance (resistor 2) and the double layer capacitance described by the CPE-Element 1 in parallel connection for description of the pseudocapacitive behavior of the Pbp at the electrode/electrolyte interphase, and (iii) an ionic resistance inside the polymer pores (resistor 3) connected with a Warburg diffusion (W_s_), both parallel to a second CPE-element 2 used to express the slow ion diffusion processes toward and inside the polymer particles and the polarization of the electrode a low frequencies (Figure 7d) [47]. The series resistance of 237.8 mΩ for Pbp@HD highlights the very good ionic conductivity of the supercapacitor and the low internal resistance of the electrode. The resistance R_2_ (5.38 Ω) was rather low, which can be attributed to an efficient charge transfer at the electrode/electrolyte interface. Both values are in accordance with the fast charge/discharge characteristics from CV and GCD and high electrical conductivity. Typically for pseudocapacitors, a value of 709 μSs^n^ and a magnitude of the exponent n of 0.76 were observed for CPE1. For resistor 3, a rather low value of 9.25 Ω was also obtained, indication of a low hinderance of the diffusion of electrolyte ions inside the polymer channels. This is in accordance with a good Warburg diffusion (575.4 Ω s^−1/2^), indicated by the 45° monotonically increasing line at frequencies below 30 Hz. Although the influence of diffusion processes on the performance seems to be lower than for the pseudocapacitance, the data from the impedance measurements explain the favorable electrochemical performance of benzopyrrole.

### 3.8. Two-Electrode Cell Electrochemical Performance

**Figure 8:**
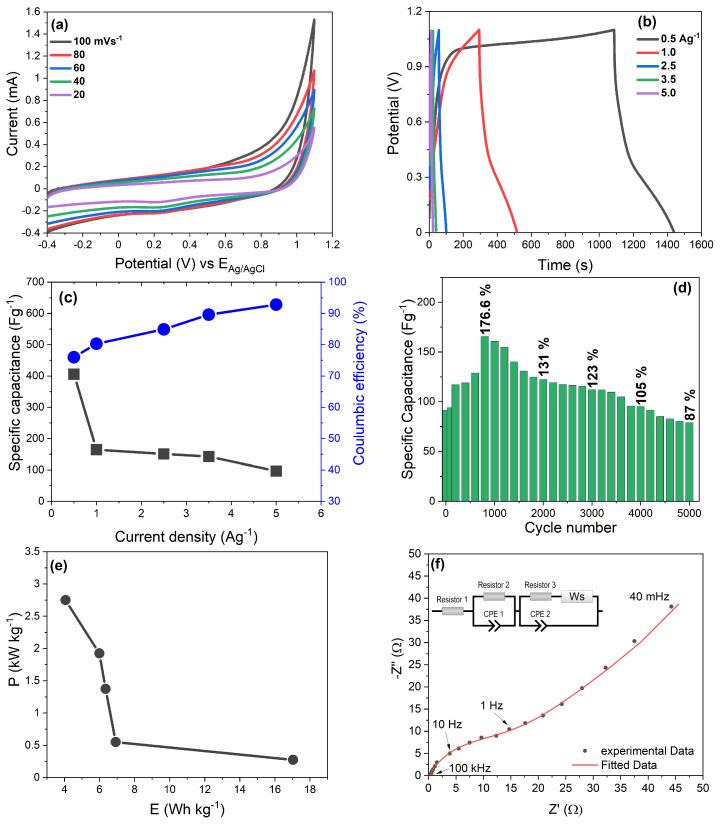
Electrochemical performance of Pbp@HD in two-electrode configuration: (**a**) CV plots at various scan rates, (**b**) GCD plots at various current densities, (**c**) specific capacitance and coulombic efficiency as a function of current density, (**d**) cycle stability at 1 A g^−1^, (**e**) Ragone plot, and (**f**) EIS at E_DC_ = 200 mV (Inset showing equivalent series circuit) for Pbp@HD.

The capacitive performance of Pbp@HD in a symmetric two-electrode configuration is assessed by measuring CV (at various scan rates) and GCD (at various current densities). As shown in Figure 8a, the CV curves were recorded in the potential window between −0.4 to 1.1 V at scan rates of 20–100 mV s^−1^. The current response of Pbp@HD increases with increments in scan rate. Furthermore, a slight deviation in the CV loop from the rectangular shape is confirming the pseudocapacitive nature of Pbp electrodes accompanied by double layer capacitance. Moreover, Pbp@HD exhibited non-triangular GCD plots at varying current densities from 0.5–5.0 A g^−1^ in the potential window of 0–1.1 V (Figure 8b). Such deviation from a perfect triangular curve is indication of the pseudocapacitive nature of Pbp@HD electrodes, which is also concluded from CV data. It is significant to note that with the rise in current density from 0.5–5.0 A g^−1^, the specific capacitance shows a decline from 405.5 ± 1.8 F g^−1^ (0.5 A g^−1^), 166.0 ± 2.0 F g^−1^ (1.0 A g^−1^), 151 ± 2.0 F g^−1^ (2.5 F g^−1^), 142.5 ± 1.5 F g^−1^ (3.5 F g^−1^), and 96.7 ± 1.0 F g^−1^ (5.0 F g^−1^) (calculated from Equation (4)). This is ascribed to the fact that at lower current densities, the electrolyte ions can deeply interact with the bulk of Pbp@HD and hence it takes a longer time for charging and discharging. On the other hand, ions interact only with the electrode surface, therefore the charge-discharge time is lesser which leads to small specific capacitance at higher current densities [48]. The results are displayed in Figure 8c.

The coulombic efficiency as a function of current density is determined from Equation (5). From Figure 8c it can be seen that the coulombic efficiency remains at about 76% at 0.5 A g^−1^ and reaches 92% when the current density is increased to 5.0 A g^−1^. The higher coulombic efficiency at high current densities is attributed to the distortable insertion/de-insertion at the interface between the Pbp@HD electrode and electrolyte at high-current densities [49]. On the other hand, the low coulombic efficiency is ascribed to the charge leakage occurring during the discharging, resulting in a decrease in discharge time. Moreover, the charging time is increased which produces excessive charge causing water oxidation at the electrode surface [50].

The electrochemical cycling stability of Pbp@HD in a symmetrical two-electrode configuration was investigated for 5000 cycles at 1 A g^−1^. As shown in Figure 8d, it was observed that the percentage capacitance retention increased to 176% during the first 1000 GCD cycles. This could be due to the structural changes that occur in the active electrode material after repeated cycles, leading to improved accessibility of ions to the available active sites. Thereafter, a decrease is observed due to intercalation/de-intercalation of ions causing volume changes during the process and resulting in a loss of redox activity [6]. A high capacitance retention of 87% after 5000 cycles indicates excellent cycling stability of Pbp@HD.

Energy density (E) and power density (P) are important in evaluating the manufactured Pbp@HD electrodes for successful application in supercapacitors. In the following, Equations (6) and (7) were used to calculate the gravimetric energy and power densities in a symmetrical two-electrode configuration. An inverse relationship between energy and power densities can be seen in the Ragone plot (Figure 8e). A maximum gravimetric energy density of 17 Wh kg^−1^ was achieved at a power density of 275 W kg^−1^ at 0.5 A g^−1^. When the power density was increased to 5.0 A g^−1^, a reduced energy density of 4.1 Wh kg^−1^ at 2.75 kW kg^−1^ was still achieved. These values are higher compared to the previously reported neat Pbp of 2.11 Wh kg^−1^ and 135 W kg^−1^ (1 Ag^−1^) [32].

Analogous to the electrochemical impedance spectroscopic three-electrode measurements, an investigation of the symmetrical Pbp@HD two-electrode cell setup was carried out. For comparability, the same electrical equivalent circuit diagram was used for the analysis of the individual parts of the processes. The Nyquist plot is shown in Figure 8f. As with the other measurement, similar impedance behavior was observed. The x-axis section describes the series resistance, an implied semicircle in the mid-frequency range contains the charge transfer resistance of the polymer, and the monotonic increase in impedance in the low frequency range is caused by long ion diffusion processes. The series resistance (resistor 1) for Pbp@HD of 269.4 mΩ remained almost unchanged compared to the three-electrode arrangement and indicates a very good ionic conductivity of the supercapacitor and the low internal resistance of the electrode. The same applies to the charge transfer resistance (resistor 2) with an excellent value of 81.1 mΩ. For CPE1 a value of 4.76 mSs^n^ with a value for the exponent n of 0.91 was obtained. The behavior describes the pseudocapacitance of the Pbp well, but the processes have a stronger capacitive influence than in the three-electrode cell design. A similar trend was found for the diffusion processes, where an ionic resistance of 15.1 Ω was found and the Warburg coefficient was much lower than in the three-electrode setup with a value of 168 Ω s^−1/2^. This indicates that the porous structure of Pbp@HD only slightly hinders the transport of ions.

In summary, the electrochemical performance of Pbp@HD electrodes in a two-electrode setup is better than previously reported supercapacitors based on pure Pbp electrodes, which are summarized in Table 2.

## 4. Conclusions

In this work we were able to demonstrate that the overall properties and performance of conjugated polymers, polybenzopyrroles in particular, can be customized via optimization of the synthetic route and reaction parameters. Polybenzopyrroles were synthesized via an efficient, facile, and simple chemical oxidative polymerization approach. A systematic variation of reactant concentrations was undertaken to identify optimized synthesis conditions for this substance class. For each polybenzopyrrole, a detailed structural, chemical, and electrochemical analysis was completed using a variety of analytical tools, i.e., FTIR, UV-Vis, XRD, SEM/EDX, TGA, CV, GCD, and EIS. The results exhibited a strong structure–performance relationship on various parameters. The polybenzopyrrole synthesized under optimized reaction conditions tended to show better electrochemical performance compared to all other polymers prsesented in this work and those synthesized using similar approaches by other research groups. A maximum specific capacitance of 166 ± 2.0 F g^−1^ at 1 A g^−1^ was obtained and a capacitance retention of 87% after 5000 cycles. In addition, high gravimetric energy density of 17 Wh kg^−1^ and power density of 2.75 kW kg^−1^ were obtained at these process conditions. The results showed that the reaction parameters mainly determine the polymer chain conjugation, charge carrier mobility along the Pbp backbone, crystallinity, thermal stability, and morphology. These properties are furthermore reflected in the electrochemical activity and stability.

This systematic approach made it possible to achieve a low cost polybenzopyrrole electrode material with moderately high specific capacitance, excellent cycling stability, and relatively high energy density and power density. Moreover, it renders easy processability of the prepared polybenzopyrrole through an uncomplicated, economical, and simple dip-coating electrode fabrication technique by eliminating toxic and costly binders. Although there is still a long way to go before a practical Faradaic supercapacitor can be developed, we are confident that it is possible to open up new avenues for low-cost Faradaic energy storage. This work provides an impetus for high quality research for the commercialization of conductive polymer-based Faradaic supercapacitors in the future.

## Figures and Tables

**Table 1:** Details of Pbp preparation by chemical oxidative polymerization under various reaction parameters and their sample designation.

No.	APS/BP (Mole Ratio)	Concentration of H_2_SO_4_ (mol L^−1^)	Amount of DBSA (mL)	Yield (%)	Sample Code ^(a)^
**Effect of APS/BP mole ratio**					
1	0.25	0.1	1.0	2.5%	S-0.25A/B
2	1.0	0.1	1.0	9.3%	S-1.0A/B
3	1.25	0.1	1.0	24.7%	S-1.25A/B
4	2.0	0.1	1.0	38.2%	S-2.0A/B
5 ^(b)^	**2.5**	0.1	1.0	55.9%	S-2.5A/B
6	2.8	0.1	1.0	15.7%	S-2.8A/B
7	3.0	0.1	1.0	3.0%	S-3.0A/B
**Effect of H_2_SO_4_ concentration**					
8	2.5	0.05	1.0	13.4%	S-0.05H
9 ^(b)^	2.5	**0.1**	1.0	55.9%	S-0.1H
10	2.5	0.5	1.0	35.0%	S-0.5H
11	2.5	1.5	1.0	24.0%	S-1.5H
12	2.5	2.0	1.0	18.6%	S-2.0H
**Effect of DBSA amount**					
13	2.5	0.1	0.5	37.2%	S-0.5D
14 ^(b)^	2.5	0.1	**1.0**	55.9%	S-1.0D
15	2.5	0.1	1.5	28.6%	S-1.5D
16	2.5	0.1	2.0	24.0%	S-2.0D

^(a)^ Sample codes with **A/B**, H, and D entitles samples with different APS/BP mole ratio, H_2_SO_4_ concentration, and amount of DBSA, respectively. ^(b)^ S-2.5A/B, S-0.1H, and S-1.0D prepared under similar reaction parameters (optimized values represented in bold).

**Table 2:** Comparison of electrochemical performance of conjugated Pbp for symmetric two-electrode supercapacitor in 1 M H_2_SO_4_.

Electrode Material	Specific Capacitance/F g^−1^	Energy Density/Wh kg^−1^	Power Density/W kg^−1^	Cycle Stability	Reference
Pure Pbp	23.8 (1 A g^−1^)	2.11 (1 A g^−1^)	135 (1 A g^−1^)	82% (after 5000 cycles at 1 A g^−1^)	[32]
Pure Pbp	117 (1 A g^−1^)	2.60 (1 A g^−1^)	-	73% (after 5000 cycles at 1 A g^−1^)	[49]
Pbp Nanospheres	36.0 (0.5 A g^−1^)	-	-	-	[51]
Pure Ppb	110 (1 A g^−1^)	3.81 (1 A g^−1^)	248 (1 A g^−1^)	78% (after 5000 cycles at 5 A g^−1^)	[52]
Pbp@HD	166 ± 2.0 (1 A g^−1^)	17.0 (1 A g^−1^) 4.10 (5 A g^−1^)	550 (1 A g^−1^) & 2750 (5 A g^−1^)	87% (after 5000 cycles at 5 A g^−1^)	This work

## Data Availability

The data presented in this study are available upon request from the corresponding author.

## Supplementary Materials

The following are available online at https://www.mdpi.com/article/10.3390/polym13172883/s1, Figure S1: Optimization of the synthesis conditions of Pbp, Figure S2: FTIR spectra of all samples, Figures S3 and S4: SEM analysis of the Pbp samples, Figures S5 and S6: XRD analysis of the Pbp samples, Figure S7: UV/Vis analysis of all Pbp samples, Figure S8: Results of the analysis of the CV data of all Pbp, Table S1: TGA results of Pbp samples, Table S2: summary of the results from CV investigation.

## References

[1] Kim J., Kim J.H., Ariga K. (2017). Redox-Active Polymers for Energy Storage Nanoarchitectonics. Joule.

[2] Gao P., Chen Z., Gong Y., Zhang R., Lui H., Tang P., Chen X., Passerini S., Lui J. (2020). The role of cation vacancies in electrode materials for enhanced electrochemical energy storage: Synthesis, advanced characterization, and fundamentals. Adv. Energy Mater..

[3] Santino L.M., Lu Y., Acharya S., Bloom L., Cotton D., Wayne A., D’Arcy J.M. (2016). Enhancing Cycling Stability of Aqueous Polyaniline Electrochemical Capacitors. J. Appl. Mater. Interfaces.

[4] Zhao C., Jia X., Shu K., Yu C., Wallace G.G., Wang C. (2020). Conducting polymer composites for unconventional solid-state supercapacitors. J. Mater. Chem. A.

[5] Chakraborty I.K., Chakrabarty N., Senapati A., Chakraborty A.K. (2018). CuO@NiO/Polyaniline/MWCNT Nanocomposite as High Performance Electrode for Supercapacitor. J. Phys. Chem. C.

[6] Rashti A., Wang B., Hassani E., Feyzbar-Khalkhali-Nejad F., Zhang X., Oh T.-S. (2020). Electrophoretic deposition of nickel cobaltite/polyaniline/rGO composite electrode for high performance all-solid-state asymmetric supercapacitors. Energy Fuels.

[7] Mudila H., Prasher P., Kumar M., Kumar A., Zaidi M.G.H., Kumar A. (2019). Critical analysis of polyindole and its composites in supercapacitor application. Mat. Renew. Sustain. Energy.

[8] Torvi A.I., Munavalli B.B., Naik S.R., Kariduraganavar M.Y. (2018). Scalable fabrication of a flexible interdigital micro-supercapacitor device by *in-situ* polymerization of pyrrole into hybrid PVA-TEOS membrane. Electrochim. Acta.

[9] Shoeb M., Mobin M., Rauf M.A., Owais M., Naqvi A.H. (2018). In vitro and in vivo antimicrobial evaluation of graphene−polyindole (gr@pin) nanocomposite against methicillin-resistant staphylococcus aureus pathogen. ACS Omega.

[10] Tebyetekerwaa M., Yanga S., Pengb S., Xua Z., Shaoa W., Pana D., Ramakrishnab S., Zhu M. (2017). Unveiling Polyindole: Freestanding As-electrospun Polyindole Nanofibers and Polyindole/Carbon Nanotubes Composites as Enhanced Electrodes for Flexible All-solid-state Supercapacitors. Electrochim. Acta.

[11] Goel S., Mazumdar N.A., Gupta A. (2011). Growth of one-dimensional polyindole nanostructures. J. Nanosci. Nanotechnol..

[12] Mudila H., Rana S., Zaidi M.G.H., Alam S. (2014). Polyindole/Graphene Oxide Nanocomposites: The Novel Material for Electrochemical Energy Storage. Fuller. Nanotub. Carbon Nanostruct..

[13] Cai Z.-J., Zhang Q., Song X.-Y. (2016). Improved Electrochemical Performance of Polyindole/Carbon Nanotubes Composite as Electrode Material for Supercapacitors. Electron. Mater. Lett..

[14] Jayakrishnan P., Ramesan M.T. (2017). Synthesis, characterization, electrical conductivity and material properties of magnetite/polyindole/poly(vinyl alcohol) blend nanocomposite. J. Inorg. Organomet. Polym..

[15] Phasuksom K., Sirivat A. (2016). Synthesis of nano-sized polyindole via emulsion polymerization and doping. Synth. Met..

[16] Phasuksom K., Prissanaroon-Ouajai W., Sirivat A. (2018). Electrical conductivity response of methanol sensor based on conductive polyindole. Sens. Actuators B.

[17] Shah A.H.A., Kamran M., Bilal S., Ullah R. (2019). Cost Effective Chemical Oxidative Synthesis of Soluble and Electroactive Polyaniline Salt and Its Application as Anticorrosive Agent for Steel. Materials.

[18] Rajesh M., Manikandan R., Kim B.C., Becuwe M., Yu K.H., Raj C.J. (2020). Electrochemical polymerization of chloride doped PEDOT hierarchical porous nanostructure on graphite as a potential electrode for high performance supercapacitor. Electrochim. Acta.

[19] Waikar M.R., Rasal A.S., Shinde N.S., Dhas S.D., Moholkar A.V., Shirsat M.D., Chakarvarti S.K., Sonkawade R.G. (2020). Electrochemical performance of Polyaniline based symmetrical energy storage device. Mat. Sci. Semicond. Proc..

[20] Mucuk Z., Karakışla M., Saçak M. (2009). Synthesis of poly (o-toluidine) in DMF/water mixture using benzoyl peroxide. Int. J. Polym. Anal. Charact..

[21] Bilal S., Gul S., Holze R., Shah A.H.A. (2015). An impressive emulsion polymerization route for the synthesis of highly soluble and conducting polyaniline salts. Syn. Met..

[22] Han D., Chu Y., Yang L., Liu Y., Lv Z. (2005). Reversed micelle polymerization: A new route for the synthesis of DBSA-polyaniline nanoparticles. Coll. Surf. A Physicochem. Eng. Asp..

[23] Shakeel I.N., Ahamed M.I., Kanchi S., Kashmery H.A. (2020). Green synthesis of ZnO nanoparticles decorated on polyindole functionalized-MCNTs and used as anode material for enzymatic biofuel cell applications. Sci. Rep..

[24] Verma C.J., Keshari A.S., Dubey P., Prakash R. (2020). Polyindole modifed g-C_3_N_4_ nanohybrids via in-situ chemical polymerization for its improved electrochemical performance. Vacuum.

[25] Oh M., Kim S. (2012). Effect of dodecyl benzene sulfonic acid on the preparation of polyaniline/activated carbon composites by in situ emulsion polymerization. Electrochim. Acta.

[26] Qui B., Wang J., Li Z., Wang X., Li X. (2020). Influence of acidity and oxidant concentration in the nanostructures and electrochemical performance of polyaniline during fast microwave-assisted chemical polymerization. Polymers.

[27] Song H., Li T., Han Y., Wang R., Zhang C., Wang Q. (2016). Optimizing the polymerization conditions of conductive polypyrrole. J. Photopolym. Sci. Technol..

[28] Han M.G., Cho S.K., Oh S.G., In S.S. (2002). Preparation and characterization of polyaniline nanoparticles synthesized from DBSA micellar solution. Synth. Met..

[29] Gahlout P., Choudhary V. (2016). Tailoring of polypyrrole backbone by optimizing synthesis parameters for efficient EMI shielding properties in X-band (8.2-12.4 GHz). Synth. Met..

[30] Djara R., Holade Y., Merzouki A., Masquelez N., Cot D., Rebiere B., Petit E., Huguet P., Canaff C., Morisset S. (2020). Insights from the physico-chemical and electrochemical screening of the potentiality of the chemically synthesized polyaniline. J. Electrochem. Soc..

[31] Ram M.S., Palaniappan S. (2004). A process for the preparation of polyaniline salt doped with acid and surfactant groups using benzoyl peroxide. J. Mater. Sci..

[32] Majumder M., Choudhary R.B., Koiry S.P., Thakur A.K., Kumar U. (2017). Gravimetric and volumetric capacitive performance of polyindole/carbon black/MoS2 hybrid electrode material for supercapacitor applications. Electrochim. Acta.

[33] Chhattise P., Handore K., Horne A., Mohite K., Chaskar A., Dallavalled S., Chabukswar V. (2016). Synthesis and characterization of Polyindole and its catalytic performance study as a heterogeneous catalyst. J. Chem. Sci..

[34] Olvera-Gracia M., Aguilar-Hernandez J.R. (2014). Conductivity and crystallinity of polyethylene oxide/polyaniline microfibers obtained by electrospinning. J. Appl. Res. Technol..

[35] Bhagat D.J., Dhokane G.R. (2015). Novel synthesis and DC electrical studies of polyindole/poly(vinyl acetate) composite films, Chem. Phys. Lett..

[36] Tebyetekerwa M., Wang X., Marriam I., Dan P., Yang S., Zhu M. (2017). Green approach to fabricate polyindole composite nanofibers for energy and sensor applications. Mat. Lett..

[37] Hassanien R., Al-Hinai M., Al-Said S.A.F., Little R., Siller L., Wright N.G., Houltom A., Horrocks B.R. (2010). Preparation and characterization of conductive and photoluminescent DNA-templetaed polyindole nanowires. ACS Nano.

[38] Bhagat D.J., Dhokane G.R. (2015). Novel photoluminescence and optical investigation of poly(vinylacetate)/polyindole composites synthesized via chromium chloride as oxidant. Appl. Surf. Sci..

[39] Liao Y., Wang X., Qian W., Li Y., Li X., Yu D.-G. (2012). Bulk synthesis, optimization, and characterization of highly dispersible polypyrrole nanoparticles toward protein separation using nanocomposite membranes. J. Coll. Interface Sci..

[40] Han Y.-G., Kusunose T., Sekino T. (2009). One-step reverse micelle polymerization of organic dispersible polyaniline nanoparticles. Synth. Met..

[41] Unal H.I., Sahan B., Erol O. (2012). Investigation of electrokinetic and electrorheological properties of polyindole prepared in the presence of a surfactant. Mat. Chem. Phys..

[42] Bilal S., Begum B., Gul S., Shah A.H.A. (2018). PANI/DBSA/H_2_SO_4_; A promising and highly efficient electrode material for aqueous supercapacitors. Synth. Met..

[43] Xu G., Wang E., Qu X., Yin Y., Chu L., He B., Wu H., Fang J., Bao Y., Liang L. (2009). Electrochemical properties of polyaniline in p-toluene sulfonic acid solution. Eur. Polym. J..

[44] Syduly B., Palaniappan S.S., Sirnivas P. (2013). Nano fibre polyaniline containing long chain and a small molecule dopants and carbon composites for supercapacitors. Electrochim. Acta.

[45] Fathi M., Shaghafi M., Mahboubi F., Mohajerzadeh S. (2014). Synthesis and electrochemical investigation of polyaniline/unzipped carbon nanotube composites as electrode material in supercapacitors. Synth. Met..

[46] Zhu D., Zhou Q., Liang A., Zhou W., Chang Y., Li D., Wu J., Ye G., Xu J., Ren Y. (2020). Two-step preparation of carbon nanotudes/RuO2/polyindole ternary nanocomposites and their application as high-performance supercapacitors. Front. Mater. Sci..

[47] Ogihara N., Itou Y., Sasaki T., Takeuchi Y. (2015). Impedance Spectroscopy Characterization of Porous Electrodes under Different Electrode Thickness Using a Symmetric Cell for High-Performance Lithium-Ion Batteries. J. Phys. Chem. C.

[48] Choudhary R.B., Ansari S., Purty B. (2020). Robust electrochemical performance of polypyrrole (PPy) and polyindole (PIn) based hybrid electrode materials for supercapacitor application: A review. J. Energy Storage.

[49] Majumder M., Choudhary R.B., Thakur A.K., Rout C.S., Gupta G. (2018). Rare earth metal oxide (RE2O3; RE = Nd, Gd, and Yb) incorporated polyindole composites: Gravimetric and volumetric capacitive performance for supercapacitor applications. New J. Chem..

[50] Wu J., Xu L., Zhou W., Jiang F., Liu P., Zhang H., Jiang Q., Xu J. (2020). Fishnet-like, nitrogen-doped carbon films directly anchored on carbon cloths as binder-free electrodes for high-performance supercapacitor. Glob. Chall..

[51] Zhou Q., Zhu D., Ma X., Mo D., Jiang F., Xu J., Zhou W. (2016). PEDOT:PSS-assisted polyindole hollow nanospheres modified carbon cloth as high performance electrochemical capacitor electrodes. Electrochim. Acta.

[52] Choudhary R.B., Majumder M., Thakur A.K. (2019). Two-dimensional exfoliated MoS2 flakes integrated with polyindole for supercapacitor application. Chem. Select.

