# Supplementary material for: Physical, Chemical, and Electrochemical Properties of Redox-Responsive Polybenzopyrrole as Electrode Material for Faradaic Energy Storage
Source: Polymers (Basel). 2021 Aug 27;13(17):2883. doi: 10.3390/polym13172883 (PMC8434118; doi:10.3390/polym13172883)
Supplement: Supplementary file 1 [file polymers-13-02883-s001.zip › polymers-1293929-supplementary.pdf]

Supplementary Information

Physical, Chemical, and Electrochemical Screening of Redox-responsive Polybenzopyrrole as Electrode Material for Faradaic Energy Storage

Bushra Begum[1], Salma Bilal*[1,2], Anwar ul Haq Ali Shah[3], Philipp Röse*[2]

[1] National Centre of Excellence in Physical Chemistry 1, University of Peshawar, 25120 Peshawar, Pakistan; bushrachemist248@gmail.com
[2] Karlsruhe Institute of Technology (KIT), Institute for Applied Materials – Electrochemical Technologies (IAM-ET), 76131 Karlsruhe, Germany
[3] Institute of Chemical Science, University of Peshawar, 25120 Peshawar, Pakistan; anwarulhaqalishah@uop.edu.pk
* Correspondence: philipp.roese@kit.edu (P.R.); salmabilal@uop.edu.pk (S.B.)

**Table of content**	**Page**
**Optimization of the synthesis conditions of polybenzopyrrole**	**2-4**
**FT-IR Spectroscopy**	**4-6**
**Analysis of the Polymer structure**	**6-8**
**XRD Analysis**	**8-10**
**Optical Spectroscopy**	**10-12**
**Thermogravimetric Analysis**	**12-14**
**Three-Electrode Cell Electrochemical Performance**	**14-15**
**References**	**15-17**

S1. Optimization of the Synthesis Conditions of Polybenzopyrrole

In general, the main role of the oxidant is to initiate the polymer chain and accelerate the polymerization rate. The nucleation of the monomer depends on the ability of the oxidant used in the reaction. However, a higher amount of oxidant can lead to overoxidation due to chain

scission and short chain formation. Therefore, it is necessary to optimize the molar ratio of oxidant to monomer.

The polymerization process with a molar ratio of 0.25 APS/BP in the presence of 0.1 M H_2SO_4 and 1.0 mL DBSA was used as the starting point for Pbp synthesis. First, the APS/BP ratio was varied. Figure S1 (a) shows the effect of the amount of benzopyrrole in terms of the APS/BP molar ratio on the percent yield of Pbp while the amount of APS is kept constant. When the APS/BP molar ratio is increased from 0.25 to 3.0, some variation in the percent yield is observed. At lower APS/BP molar ratio (0.25 and 1.0), negligible amount of polymer is formed, which is due to the presence of excess benzopyrrole that reduces the efficiency of APS. It is assumed that the small amount of APS led to a successive decrease in the formation of the monomeric radical cations, resulting in a decrease in the percentage yield of Pbp. The curve shows the attainment of a maximum (56%) in the percent yield at APS/BP =2.5 (2.80 mmol benzopyrrole). This is due to the formation of gradual, elongated, and regular Pbp chains, which reach their optimum shape under the prevailing conditions of monomer and oxidant.

Above 2.5, a further increase in the APS/BP molar ratio negatively affected the percent yield. A very low percent yield (3%) of the polymer was observed for APS/BP molar ratio=3.0. The possible explanations are as follows; it is expected that the decreasing trend at higher APS/BP is a consequence of the presence of too much APS compared to benzopyrrole. A large amount of oxidant compared to benzopyrrole could cause overoxidation of the radical cations of the monomer, leading to a decrease in the chain length and growth rate of the polymer. In addition, it could also be a reason that too much APS generates a large amount of monomeric active sites that form oligomers and short conjugated Pbp chains that are highly soluble in the polymerization mixture, making it difficult for Pbp to precipitate out of the polymerization medium [1-7].

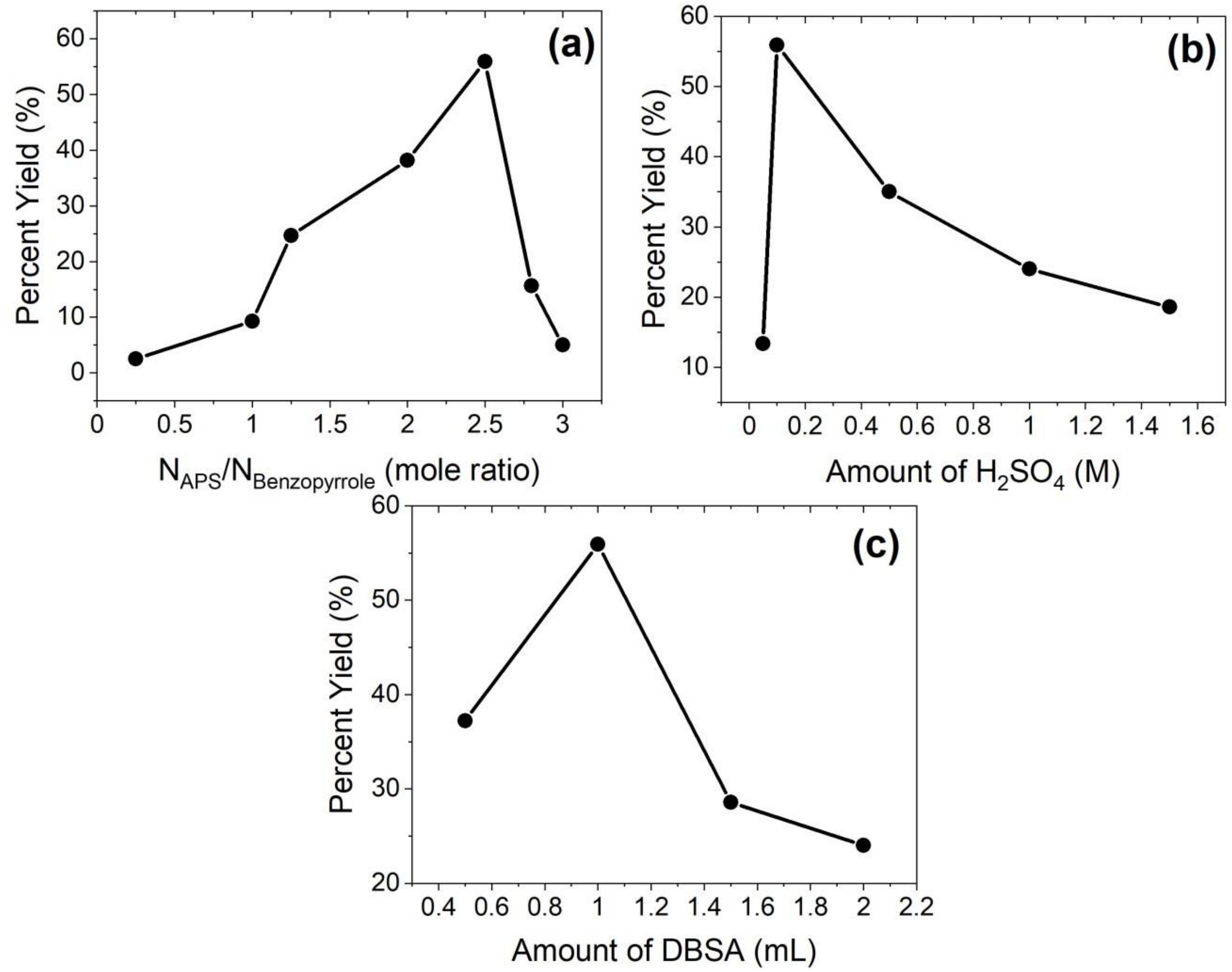

Figure S1. Influence of various reaction parameters on the percent yield of Pbp; (a) APS/BP mole ratio (from 0.25 to 3.0) at 0.1 M H_2SO_4 and 1.0 mL DBSA, (b) amount of H_2SO_4 (from 0.05 to 2.0 M) at 2.5 APS/BP ratio and 1.0 mL DBSA, and (c) amount of DBSA (from 0.5 to 2.0 mL) at 0.1 M H_2SO_4 and APS/BP mole ratio = 2.5.

In the second series of experiments, different Pbp samples were prepared with varying H2SO4 concentrations, and a constant APS/BP molar ratio of 2.5. The corresponding effect on the percentage yield of Pbp is shown in Figure S1 (b). With the change of acid concentration from 0.05 to 0.1 M, the percent yield first increases to a maximum value of 56% and then strongly decreased with further increase to 2.0 mol (6.5%). To interpret this phenomenon, we agreed that with increasing concentration, there is a prolonged polymer chain conformation due to the electrostatic repulsion of similarly charged units in the polymer chains. On the contrary, at higher H_2SO_4 concentration, excessive doping occurs. This stimulates polymer hydrolysis, which in turn reduces NH_2^+ in the chain and leads to broken and short-chain Pbp/side products that are soluble

in the polymerization medium instead of precipitating out [2, 7]. Thus, 0.1 M H_2SO_4 was the optimum combination for the selected conditions of APS/BP mole ratio and DBSA.

Finally, the amount of H_2SO_4 (0.1 M) and the APS/BP molar ratio (2.5) were kept constant, and the amount of DBSA was varied from 0.5 to 2.0 mL. The percent yield first increases (56%) and then decreases (28%) with the variation of the amount of DBSA, as shown in Figure S1 (c). The increasing trend in yield is attributed to the insertion of many bulky DBSA counterions into the Pbp backbone. However, this effect is compromised with a further increase in the amount of DBSA, which could be due to the formation of many micelles in the reaction mixture that reduce the interaction between monomers and DBSA. Therefore, the percentage yield of the polymer decreases. [3, 8]. The outcomes of this study show good endorsement with the previous studies reported for other similar conducting polymers [3-8].

S2. FT-IR Spectroscopy

The FT-IR spectra of all Pbp samples with varying reaction parameters are presented in Figure S2. Upon altering the APS/BP mole ratio, all three Pbp polymers exhibited nearly same IR vibration spectra (Figure S2a-b). However, little irregularity in peaks intensities and positions for CH out-of-plan deformation (730→728→733 cm^{-1}), NH stretching (3168→3167→3177 cm^{-1}), CH stretching of alkyl chain (2921→2920→2924 & 2850→2851→2858 cm^{-1}) and aromatic ring (1149→1148→1176 cm^{-1}) could be found with the increase mole ratio of APS/BP. These prominent red-shifts are expected due to enhanced conjugation in Pbp chain up to a 2.5 mole ratio followed by blue-shifts due to over oxidation at 2.8 mole ratio. Moreover, some peaks appeared in the range of 1702-2300 cm^{-1} which became more prominent in S-2.8A/B spectrum (Figure S2 (a) inset). The appearance of such peaks confirms the over-oxidation of the polymer due to presence of excessive APS amount than necessary for polymerization of benzopyrrole. Similar effect of oxidant to monomer mole ratio on Pbp and polyaniline was reported earlier [9]. Hence, 2.5 APS/BP mole ratio was selected as the best compromise, also with regard to the synthesis results.

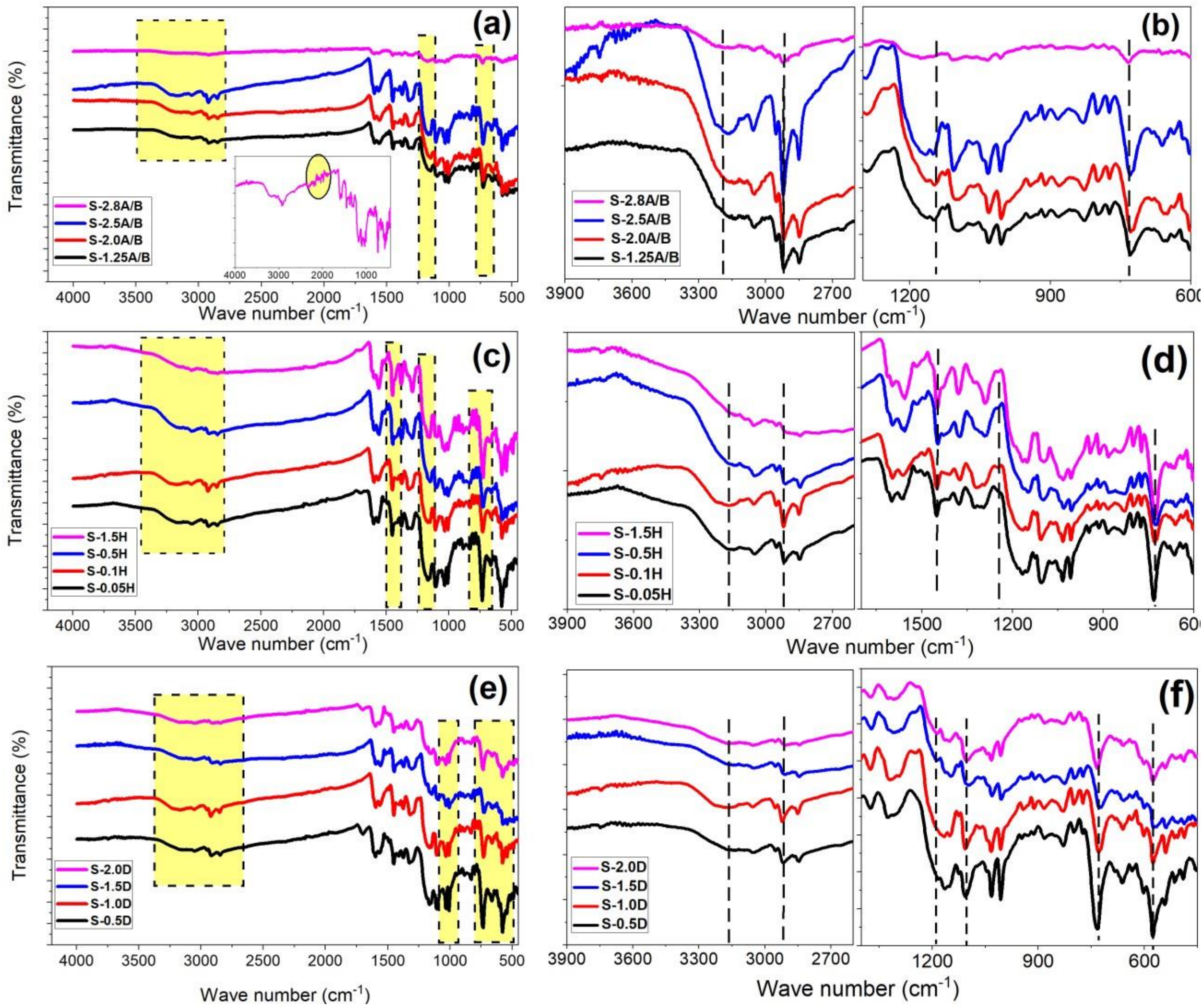

Figure S2. (a), (c), (e) FTIR spectra and (b), (d), (f) enlarged view of yellow highlighted spectral regions for Pbp samples polymerized under different reaction parameters. Inset (in a); Rescaled FTIR of S-2.8.

FT-IR spectra of Pbp with varying contents of H_2SO_4 are displayed in Figure S2c-d. The IR spectra revealed slight difference in peak position and intensities of HSO_4^{1-} (1174→1156→1170 cm^{-1}) alongwith NH stretching (3193→3164 cm^{-1}). In addition, a slight shift is also observed for CN stretching (1451→1447→1448 cm^{-1}), and CH stretching of alkyl chain (2918→2920→2928 & 2851→2850→2862 cm^{-1}). Besides, the characteristics bands (CN, NH, and CH stretching) weakened with the increase in acid concentration and almost disappeared in S-1.5H. From the observed red-shifts in these bands, particularly in HSO_4^{1-} and NH stretching peaks is attributed to the strong interaction between Pbp backbone compensating the charge of N^+. Moreover, the red-shift in CH stretching suggests higher conjugation with the addition of H_2SO_4 concentration

till 0.1 M and then decreases at 1.5 M. Our results were in good agreement with the literature [9, 10]. Besides, these results coincide with our percent yield study.

Figure S2e-f shows FT-IR spectra of Pbp with varying amounts of DBSA. The IR spectra peaks shifted a little towards lower and then higher wave number with the increase in DBSA content. The following peak heights and positions changed; SO_3^{1-} (576→573 cm^{-1}) alongwith CH out-of-plan deformation (734→728→733 cm^{-1}), NH stretching (3171→3167→3179 cm^{-1}), and CH stretching of alkyl chain (2916→2911 & 2851→2846 cm^{-1}). Contrary to the expectation, the peaks seem to be weakened in FTIR spectrum of S-2.0D than Pbp with lower DBSA amount. This might be due to steric hinderance offered by the presence of large amount of DBSA molecules, thus making it difficult for DBSA anion to interact with Pbp chain [11]. Henceforth, FTIR spectra signify the degree of conjugation and sensitivity of structural characteristics of developed well-doped Pbp samples to the various reaction parameters.

S3. Analysis of the Polymer Structure

Figure S3 presents SEM pictures for Pbp samples with varying concentrations of H_2SO_4. Interestingly, upon altering the concentration of H_2SO_4 (from 0.05 to 0.1 M), the Pbp microparticles demonstrated a lower size with the less irregular agglomeration. However, further increment in the amount of acidity resulted into large, aggregated particles. This might result in the formation of polarons/bipolarons, producing large particles. A study on the effect of HCl concentration on the morphology of polyaniline also concluded similar effect [12]. Thus, H_2SO_4 with 0.1 M concentration was selected as the optimized value.

As shown in Figure S4, Pbp revealed a drastic change in morphology with DBSA amount. The irregularity and roughness of Pbp surface appears to be decreased (S-0.5D to S-1.0D) and then increased (S-1.5D to S-2.0D) with the increase in DBSA amount. Moreover, the polymer becomes more compact as can be seen from the SEM of S-2.0D with highest amount of DBSA (2.0 mL). This phenomenon might also be attributed to the surfactant's micelle fusion due to high amount of DBSA [13, 14].

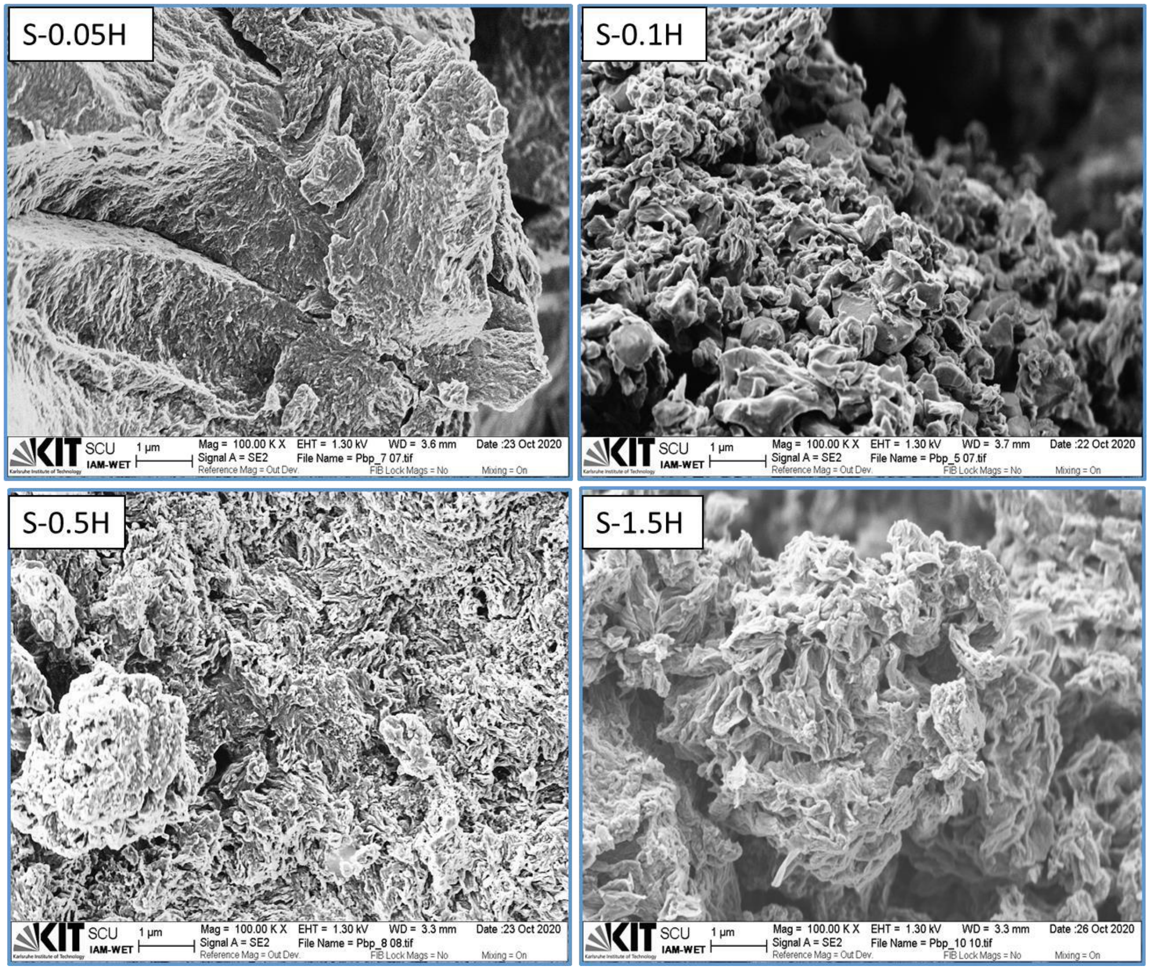

Figure S3. SEM of Pbp samples with various H_2SO_4 concentrations from 0.05 M to 1.5 M (1.0 mL DBSA, APS/BP mole ratio = 2.5).

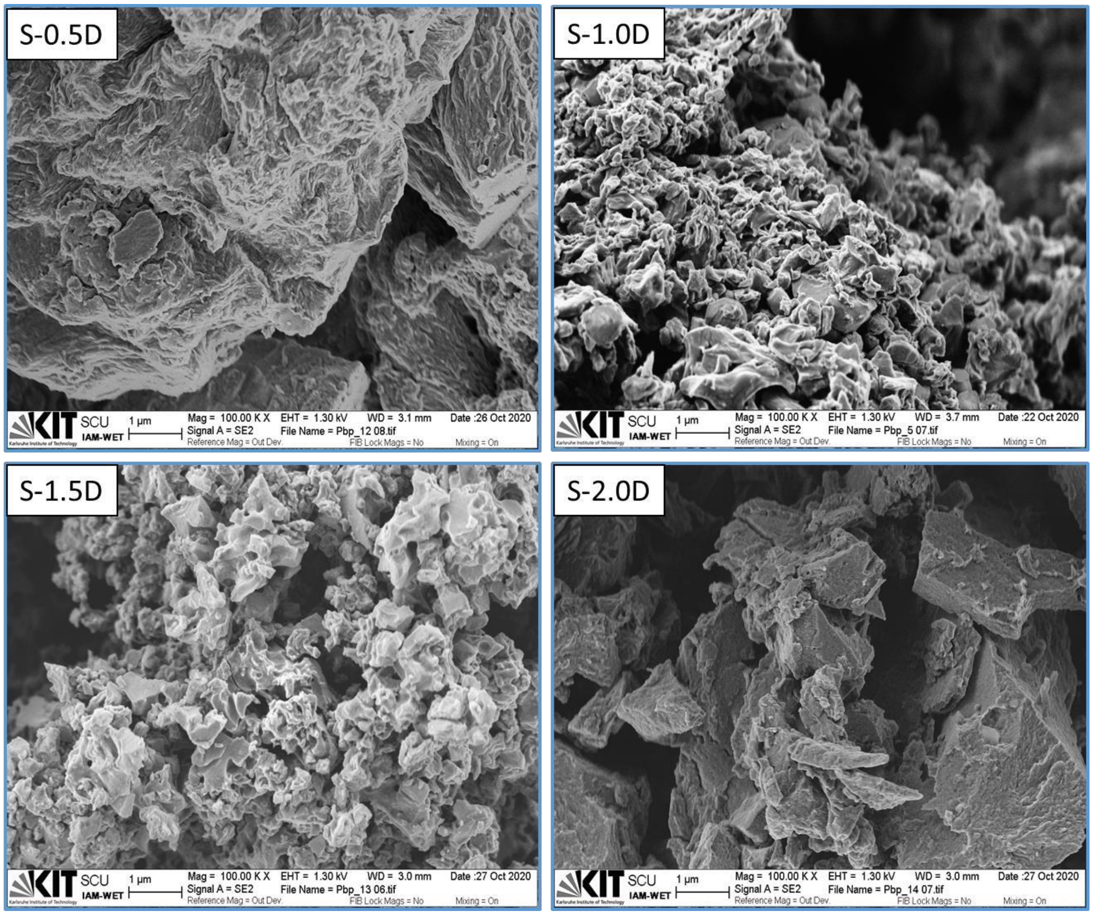

Figure S4. SEM of Pbp samples with different DBSA amounts, 0.5 to 2.0 mL (0.1M H_2SO_4, APS/BP mole ratio = 2.5).

S4. XRD Analysis

Figure S5 shows the XRD spectra of Pbp samples (S-0.05H-S-1.5H) with different concentrations of H_2SO_4. These patterns also show a prominent broad hump at $2\theta = 17.2$-$18.0°$ and a sharp peak $2\theta \sim 26.5$-$26.9°$. Besides, extra short peaks are also observed at various 2θ values. With varying H_2SO_4 these peaks 2θ values as well as intensity changed which implies that the structural changes occurred due to acid concentration. Also, the disappearance of short peaks in the S-1.5H sample is ascribed to the less ordered and short conjugated polymer chains due to high H_2SO_4 concentration which might results into reduced electro-activity of the polymer. A similar effect of higher acidity on the other conjugated polymers, polypyrrole and polyaniline, was also observed by other researchers [9, 15].

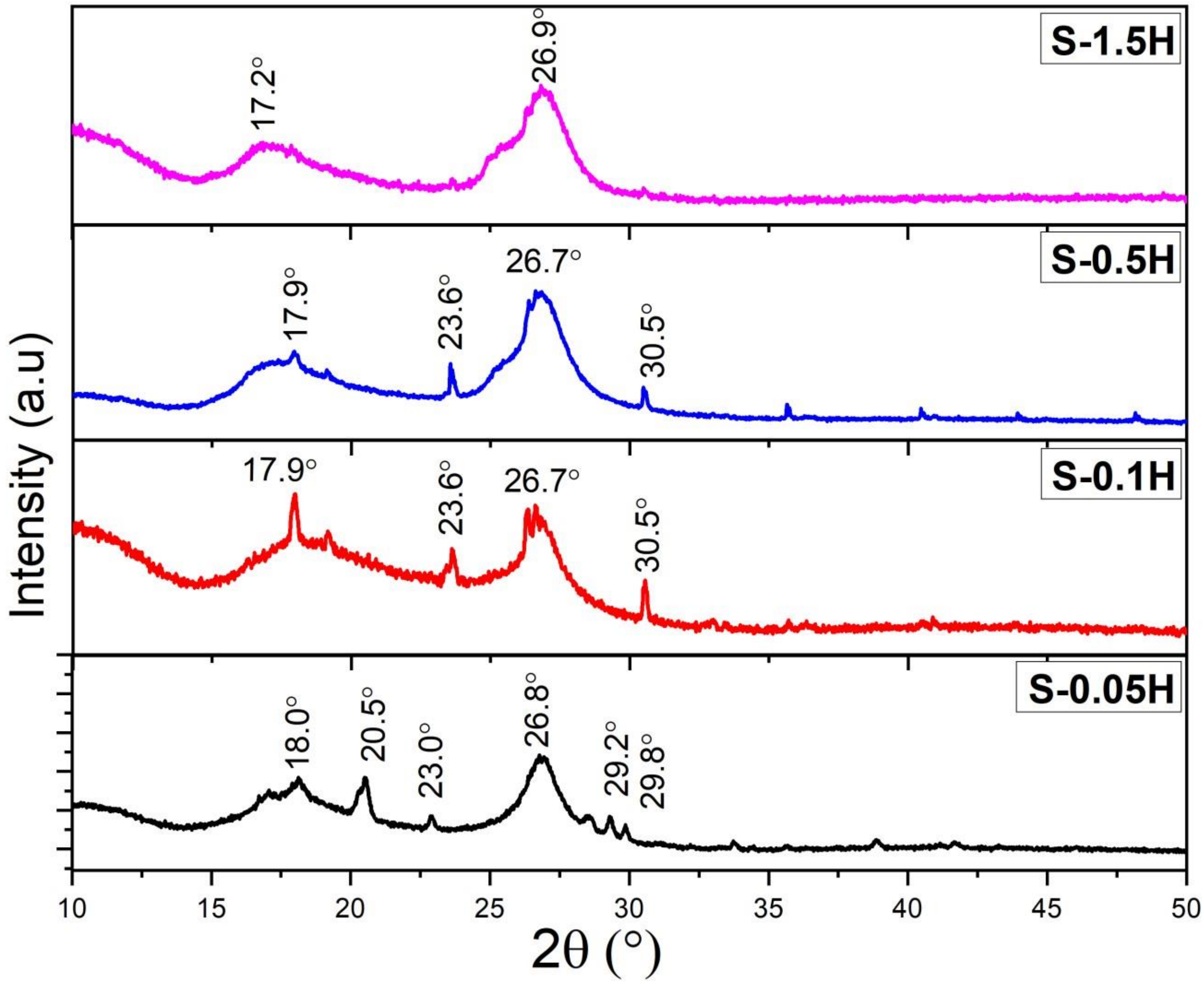

Figure S5. XRD patterns of Pbp samples prepared with 0.05 to 1.5M H_2SO_4 concentrations (1.0 mL DBSA, APS/BP mole ratio = 2.5).

The effect of DBSA amount (from 0.5 to 2.0 mL) on XRD of Pbp samples is shown in Figure S6. The pattern of these samples presents signature broad and sharp peak in the $16° < 2θ < 27°$. As mentioned above, the peaks related to DBSA long chain seems to be enhanced in the samples S-0.5D to S-1.5D with the increase of DBSA amount. Hence, it is said that the molecular arrangement in these polymer samples become more ordered and increased their crystallinity. However, these peaks disappeared on further increase in DBSA (as shown in S-2.0D). The possible reason for this might be the presence of higher amount of DBSA counter ions relative to the Pbp molecules which formed micelles and retarded diffraction peaks. These results agree with the earlier reported literature [13].

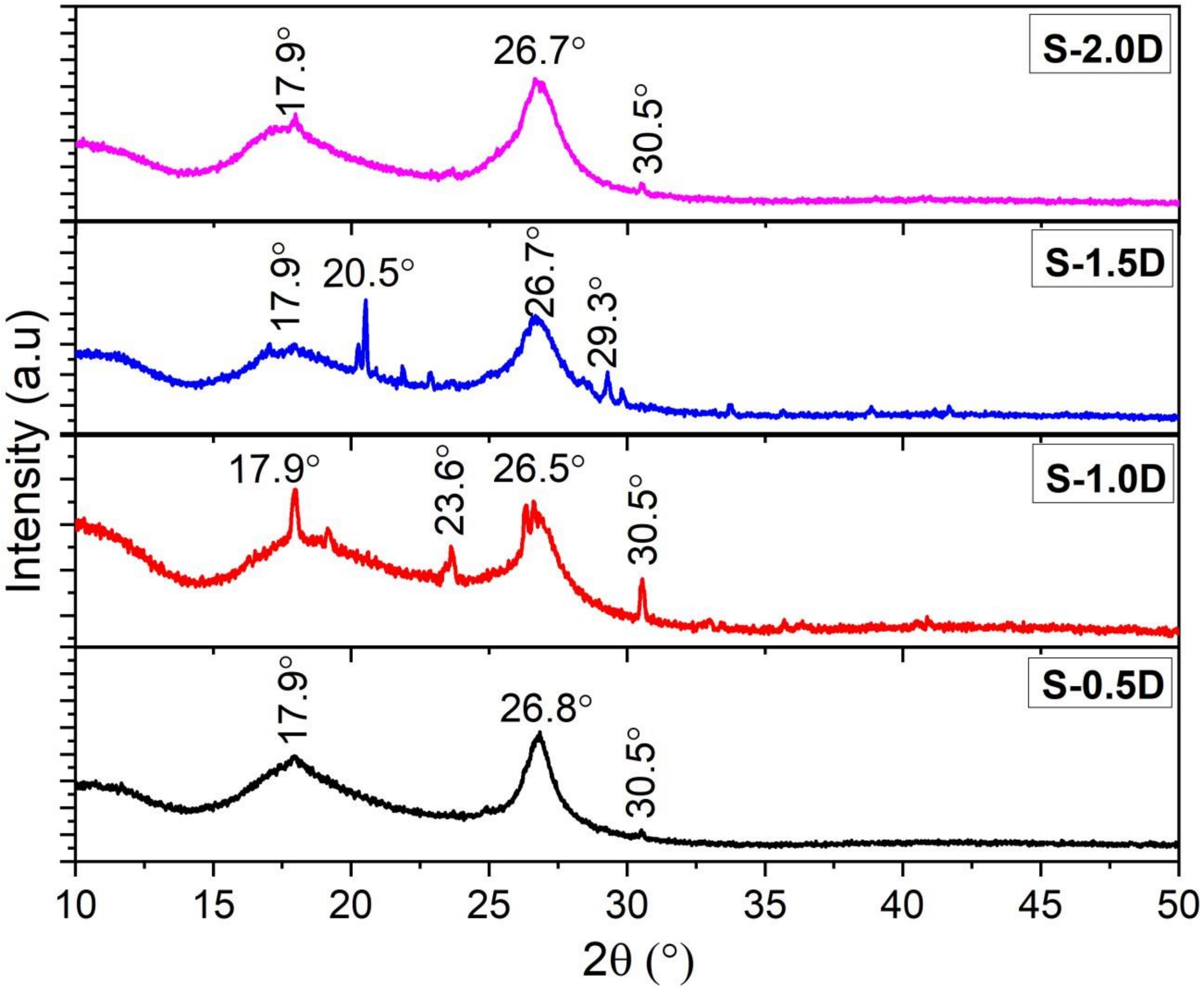

Figure S6. XRD patterns of Pbp samples as a function of DBSA amounts (0.5-2.0 mL) at 0.1M H_2SO_4, APS/BP mole ratio = 2.5.

S5. Optical Spectroscopy

With the increased APS/BP mole ratio from 1.25 to 2.5, the pi-pi* optical absorption band slightly shifted from 272 to 274 for S-1.25A/B-P-2.5A/B (Figure S7 (a-b)). Further, the increase in APS/BP mole ratio to 2.8 resulted in the blue-shift of pi-pi* absorption band towards 273 nm for S-2.8A/B. This confirms the decrease in delocalization owing to polymer chains splitting into shorter chains in the presence higher APS amount with respect to benzopyrrole [16].

Figure S7 (c-d) shows the effect of H_2SO_4 concentration on ultraviolet-visible spectra of Pbp. As explained earlier, H_2SO_4 concentration also influence the conjugated chain length and percent yield of Pbp. pi-pi* transition band revealed a red-shift from 261-274 nm with the increase

in H_2SO_4 concentration from 0.05-0.1 M (S-0.05H-S-0.1H). This corresponds to extensive conjugated Pbp chain formation. However, a decrease conjugated polymer chains length due to over-oxidation is confirmed from the blue-shift in pi-pi* band to 268 nm till 1.5 M H_2SO_4 concentration (S-1.5H). Similar shifts are also observed in other absorption bands. However, the polaronic excitation band is enhanced demonstrating a higher doping of H_2SO_4. The effect of acidity on absorption spectra of conjugated polymers are already reported in literature [9, 17, 18]. From these spectral results, the optimum H_2SO_4 concentration was selected as 0.1 M.

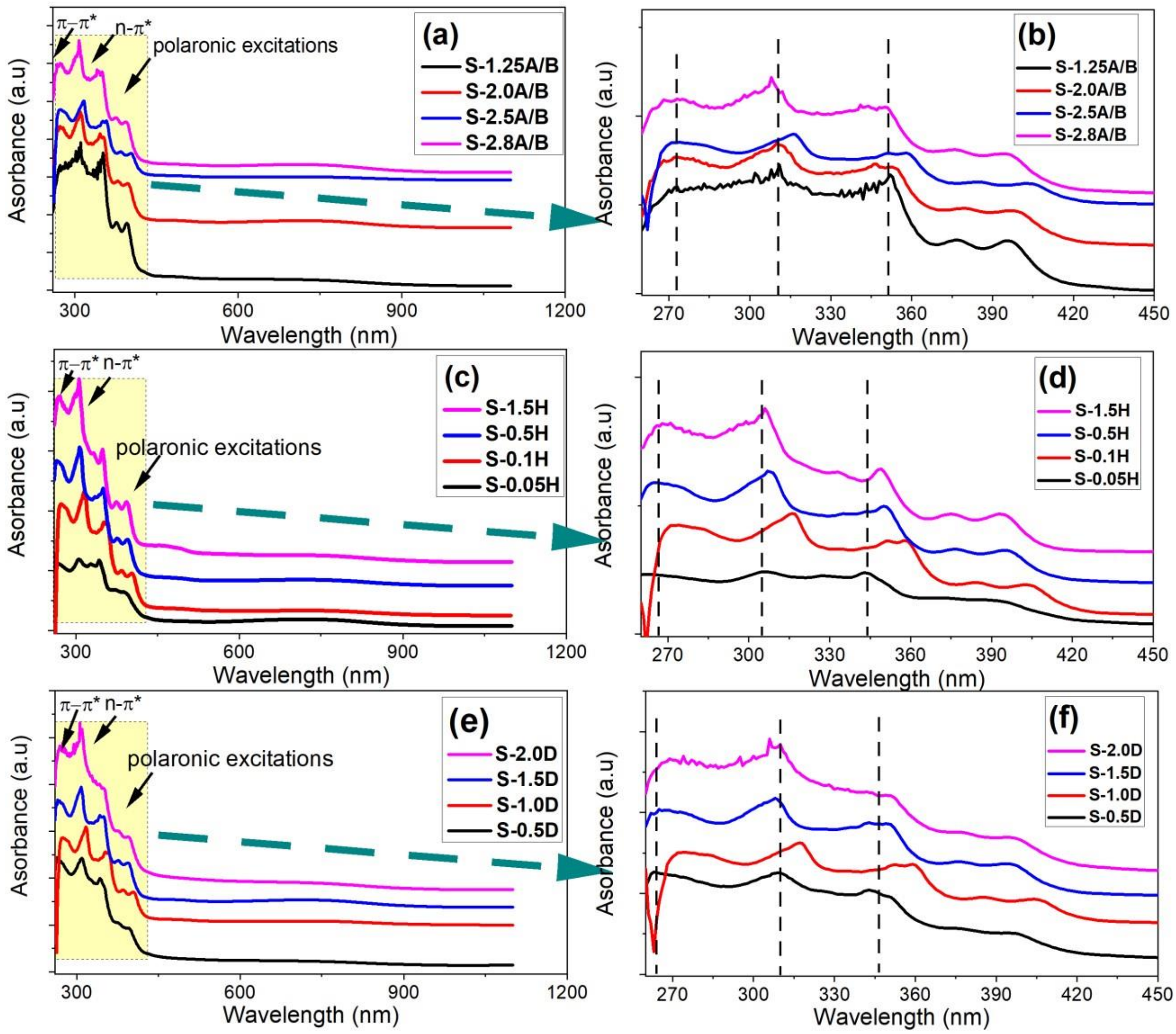

Figure S7. Ultraviolet-visible absorption spectra of Pbp as a function of (a) APS/BP mole ratio, (c) concentration of H_2SO_4, (e) amount of DBSA, and (b), (d), (f) are corresponding zoomed spectra of lower wavelength region.

Ultraviolet-visible absorption spectra of Pbp polymerized with different DBSA amounts is shown in Figure S7 (e-f). These spectra display a very similar transitions bands as observed for Pbp prepared with varying APS/BP mole ratio and H_2SO_4 concentration. At lower DBSA amount (0.5 mL), pi-pi* transition band is observed at 262 nm for S-0.5D. With the introduction of higher amounts of DBSA, pi-pi* transition peak first gets a red-shift of to 274 nm (increased conjugation) and then blue shift to 270 nm (reduced conjugation) is observed. Besides, polaronic bands also shifts towards longer and then shorter wavelengths with DBSA amount [18]. Due to higher red-shift, S-1.0D with 1.0 mL DBSA was selected as the optimum amount of DBSA under the prevailing reaction conditions.

S6. Thermogravimetric Analysis

The thermal degradation study of Pbp polymers with various APS/BP mole ratio are shown in Table S1. The weight loss of 14.2%, 11.6%, 10.3%, and 11.7% is observed S-1.25A/B, S2.0A/B, S-2.5 A/B, and S-2.8A/B, respectively. Next, elimination of dopants (H_2SO_4 & DBSA) occurred with mass loss of 32% (S-1.25A/B), 40% (S-2.0A/B), 32% (S-2.5 A/B), and 32% (S-2.8A/B). The final mass loss of Pbp samples were as observed; 38.5% (S-1.25A/B), 43% (S2.0A/B), 50% (S-2.5A/B), and 50% (S-2.8A/B). Thus, the thermal degradation temperature of Pbp first increases slightly with APS/BP mole ratio (up-to 2.5), however, beyond this thermal stability was drastically decreased at APS/BP mole ratio = 2.8. By comparing these values, the order of highest relative thermal stability was: S-2.5A/B > S-2.0A/B > S-1.25A/B > S-2.8A/B. These findings were consistent with the already reported literature [19].

In the 2nd stage, Pbp polymers synthesized with different H_2SO_4 concentrations showed a significant mass loss 11.3%, 10.3%, 18.5%, and 19.4%, was observed for S-0.05H, S-0.1H, S-0.5H, and S-1.5H, respectively. The removal of H_2SO_4 and DBSA from S-0.05H, S-0.1H, S-0.5H, and S1.5H resulted in the corresponding mass loss of 11.3%, 10.3%, 18.5%, and 19.4%. The polymeric backbone degradation with accompanying mass loss of samples were as observed; 45.8% (S0.05H), 32% (S-0.1H), 18.8% (S-0.5H), and 21.5% (S-1.5H). From these results, it is suggested that the most thermally stable Pbp sample was S-0.1H followed by S-0.05H, S-0.5H, and then S-1.5H.

Table S1. TGA results of Pbp samples.

Sample	Degradation Temperature (°C) at various steps				Char Yield (%)
	1st step	2nd step	3rd step	4th step	
**Effect of APS/BP mole ratio**					
S-1.25A/B	49-83.9	179.8-378.6	378.6-547.2	547.2-698.6	11.8
S-2.0A/B	49-96.4.0	199.2-362.9	362.9-548.0	548.0-705.6	0.0
S-2.5A/B	49-156.6	259.8-379.8	379.8-549.9	549.9-729.1	1.82
S-2.8A/B	49-110.6	238.0-376.6	376.6-535.3	535.3-744.5	0.36
Effect of H_2SO_4 concentration					
S-0.05H	49-89.3	236-360.3	360.3-548.4	548.4-703.6	-0.5
S-0.1H	49-156.6	259.8-379.8	379.8-549.9	549.9-729.1	1.82
S-0.5H	49-140.5	214.6-402.6	402.6-534.3	534.3-746.7	0.0
S-1.5H	49-134.3	180.2-365.3	365.3-536.2	536.2-730.0	2.9
Effect of DBSA amount					
S-0.5D	49-78.9	160-360.1	360.1-545.3	545.3-710.6	1.3
S-1.0D	49-156.6	259.8-379.8	379.8-549.9	549.9-729.1	1.82
S-1.5D	49-90.2	259.2-370.9	370.9-513	513-712.6	9.11
S-2.0D	49-102.0	174.13-462.6	462.6-546.3	546.3-711.2	1.59

As seen from the Table S1, TGA of Pbp samples synthesized with different DBSA amounts revealed a percent weight loss of 7.1%, 10.3%, 11.9%, and 23.9% for corresponding samples; S-0.5D, S-1.0D, S-1.5D, and S-2.0D samples in the 2nd step. The mass loss was found to be 40.2%, 32%, 21.5%, and 13.9% for Pbp samples (S-0.5D to S-2.0D). The polymeric backbone of Pbp samples (S-0.1D to S-2.0D) exhibited a prominent mass loss of 46.2%, 50%, 50.8%, and 54.9%, respectively. Thus, it was concluded that S-1.0D was relatively more thermally stable than other samples S-2.0D > S-0.5D > S-1.5D.

S7. Three-Electrode Cell Electrochemical Performance

Table S2. Electrochemical analysis of Pbp electrodes at 100 mV s^{-1} in 1 M H_2SO_4 electrolyte using 3-electrode set-up.

Sample	Peak Potential (mV)				Charge (mC)
	I_{Ox}	II_{Ox}	I_{Red}	I_{Red}	
Effect of APS/BP mole ratio					
S-1.25A/B	45.9	959.6	-171.3	792.5	24.23
S-2.0A/B	35.8	935.8	-250.5	800.6	28.86
S-2.5A/B	26.67	860.4	-200.0	796.7	**38.95**
S-2.8A/B	101.5 broad hump	967.4	-61.3 broad hump	698.1	7.96
Effect of H_2SO_4 concentration					
S-0.05H	83.94	940.4	-283.9	843.1	11.57
S-0.1H	26.67	860.4	-200.0	796.7	**38.95**
S-0.5H	-55.08	925.5	-210.6	782.9	35.58
S-1.5H	89.6	900.9	-188.7	740.6	17.64
Effect of DBSA amount					
S-0.5D	86.89	941.6	-243.6	833.3	20.03
S-1.0D	26.67	860.4	-200.0	796.7	**38.95**
S-1.5D	239.6 broad peak		239.6 broad peak		12.10
S-2.0D	516.8 broad peak		239.6 broad peak		11.44

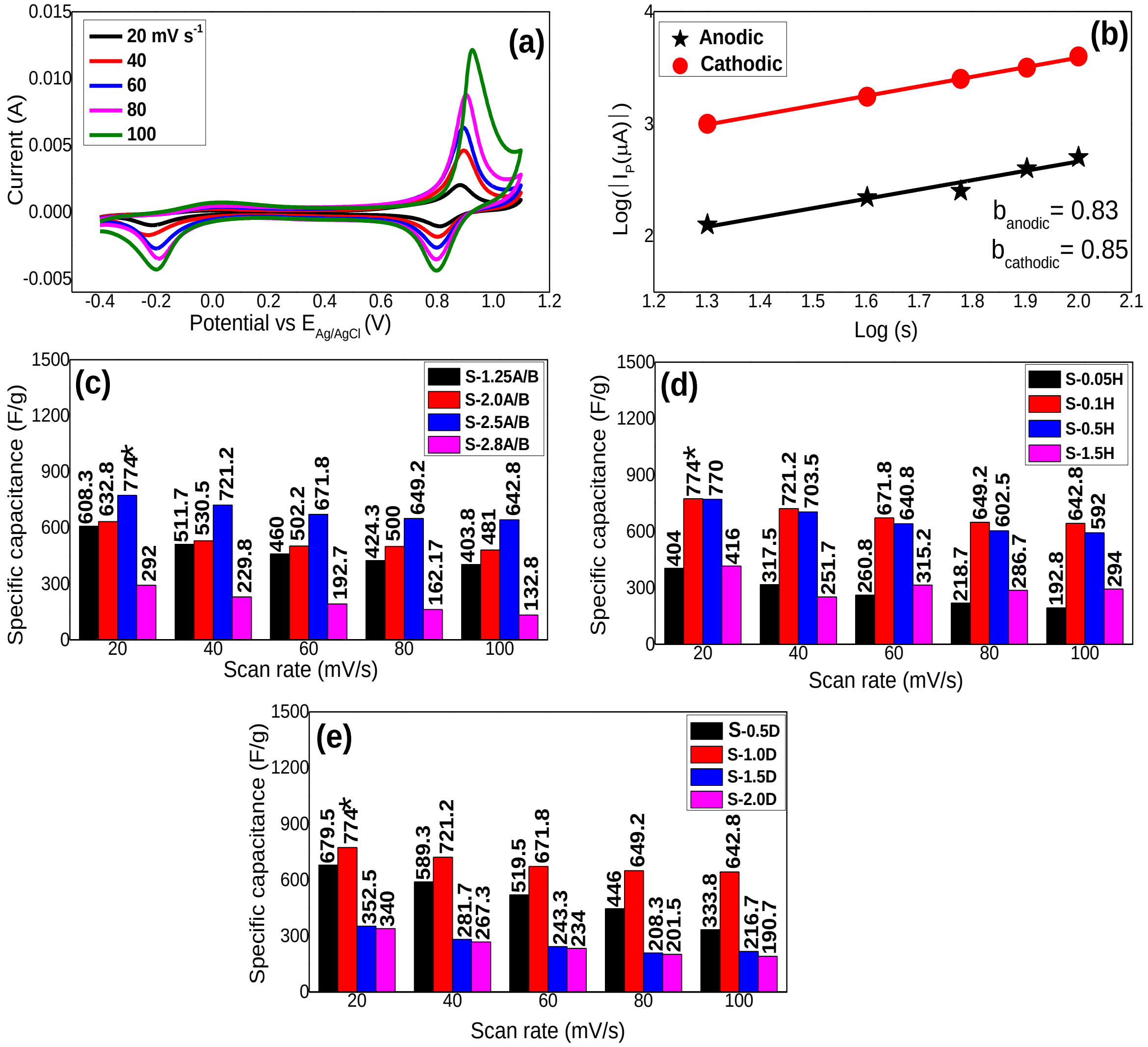

Figure S8. (a) CV curves, (b) Log(I_P) vs Log(s), and (c), (d), and (e) specific capacitance of Pbp electrodes obtained 1 M H_2SO_4 at various potential sweep rates.

References

1. A. Rashti, B. Wang, E. Hassani, F. Feyzbar-Khalkhali-Nejad, X. Zhang, T.-S. Oh, Electrophoretic deposition of nickel cobaltite/polyaniline/rGO composite electrode for high performance all-solid-state asymmetric supercapacitors. *Energy Fuels* **2020**, *34*, 6448.
2. H. Mudila, P. Prasher, M. Kumar, A. Kumar, M. G. H. Zaidi, A. Kumar, Critical analysis of polyindole and its composites in supercapacitor application. *Mat. Renew. Sus. Energ.* **2019**, *8*, 9.
3. A.H.A. Shah, M. Kamran, S. Bilal, R. Ullah, Cost Effective Chemical Oxidative Synthesis of Soluble and Electroactive Polyaniline Salt and Its Application as Anticorrosive Agent for Steel. *Materials* **2019**, *12*, 1527.

4. M. Rajesh, R. Manikandan, B. C. Kim, M. Becuwe, K. H. Yu, C. J. Raj, Electrochemical polymerization of chloride doped PEDOT hierarchical porous nanostructure on graphite as a potential electrode for high performance supercapacitor. *Electrochim. Acta* **2020**, *354*, 136669.

5. M. R. Waikar, A. S. Rasal, N. S. Shinde, S. D. Dhas, A. V. Moholkar, M. D. Shirsat, S. K. Chakarvarti, R. G. Sonkawade, Electrochemical performance of Polyaniline based symmetrical energy storage device. *Mat. Sci. Semicond. Proc.* **2020**, *120*, 105291.

6. Z. Mucuk, M. Karakışla, M. Saçak, Synthesis of poly (o-toluidine) in DMF/water mixture using benzoyl peroxide. *Int. J. Polym. Anal. Charact.* **2009**, *14*, 403.

7. S. Bilal, S. Gul, R. Holze, A.H.A. Shah, An impressive emulsion polymerization route for the synthesis of highly soluble and conducting polyaniline salts. *Synthetic Metals* **2015**, *206*, 131.

8. D. Han, Y. Chu, L. Yang, Y. Liu, Z. Lv, Reversed micelle polymerization: an easy route for the synthesis of DBSA-polyaniline nanoparticles. *Coll. Surf. A: Physicochem. Eng. Aspects* **2005**, *259*, 175.

9. B. Qui, J. Wang, Z. Li, X. Wang, X. Li, Influence of acidity and oxidant concentration in the nanostructures and electrochemical performance of polyaniline during fast microwave-assisted chemical polymerization, *Polymers* **2020**, *12*, 310.

10. H. Song, T. Li, Y. Han, R. Wang, C. Zhang, Q. Wang, Optimizing the polymerization conditions of conductive polypyrrole, *J. Photopolym. Sci. Technol.* **2016**, *29*, 803.

11. M. G. Han, S. K. Cho, S. G. Oh, S. S. In, Preparation and characterization of polyaniline nanoparticles synthesized from DBSA micellar solution. *Synthetic Metals* **2002**, *126*, 53.

12. R. Djara, Y. Holade, A. Merzouki, N. Masquelez, D. Cot, B. Rebiere, E. Petit, P. Huguet, C. Canaff, S. Morisset, T. W. Napporn, D. Cornu, S. Tingry, Insights from the physico-chemical and electrochemical screening of the potentiality of the chemically synthesized polyaniline. *J. Electrochem. Soc.* **2020**, *167*, 066503.

13. M. Oh, S. Kim, Effect of dodecyl benzene sulfonic acid on the preparation of polyaniline/activated carbon composites by in situ emulsion polymerization. *Electrochim. Acta* **2012**, *59*, 196.

14. P. Gahlout, V. Choudhary, Tailoring of polypyrrole backbone by optimizing synthesis parameters for efficient EMI shielding properties in X-band (8.2-12.4 GHz). *Synthetic Metals* **2016**, *222*, 170.

15. M. Majumder, R. B. Choudhary, S. P. Koiry, A. K. Thakur, U. Kumar, Gravimetric and volumetric capacitive performance of polyindole/carbon black/MoS_2 hybrid electrode material for supercapacitor applications. *Electrochim. Acta* **2017**, *248*, 98.

16. M. Tebyetekerwa, X. Wang, I. Marriam, P. Dan, S. Yang, M. Zhu, Green approach to fabricate

polyindole composite nanofibers for energy and senser applications. *Mat. Lett.* **2017**, *209*, 400.

17. Y. Liao, X. Wang, W. Qian, Y. Li, X. Li, D.-G. Yu, Bulk synthesis, optimization, and characterization of highly dispersible polypyrrole nanoparticles toward protein separation using nanocomposite membranes. *J. Coll. Inter. Sci.* **2012**, *386*, 148.
18. Y.-G. Han, T. Kusunose, T. Sekino, One-step reverse micelle polymerization of organic dispersible polyaniline nanoparticles, *Synthetic Metals* **2009**, *159*, 123.
19. A. Yussuf, M. Al-Saleh, S. Al-Enezi, G. Abraham, Synthesis and characterization of conductive polypyrrole: The influence of the oxidants and monomer on the electrical, thermal, and morphological properties. *Int. J. Polym. Sci.* **2018**, 1.
